# Facet Engineering of Advanced Electrocatalysts Toward Hydrogen/Oxygen Evolution Reactions

**DOI:** 10.1007/s40820-023-01024-6

**Published:** 2023-02-16

**Authors:** Changshui Wang, Qian Zhang, Bing Yan, Bo You, Jiaojiao Zheng, Li Feng, Chunmei Zhang, Shaohua Jiang, Wei Chen, Shuijian He

**Affiliations:** 1 International Innovation Center for Forest Chemicals and Materials, Co-Innovation Center of Efficient Processing and Utilization of Forest Resources, Nanjing Forestry University, Nanjing, 210037 People’s Republic of China; 2 Key Laboratory of Material Chemistry for Energy Conversion and Storage, Ministry of Education, Hubei Key Laboratory of Material Chemistry and Service Failure, School of Chemistry and Chemical Engineering, Huazhong University of Science and Technology (HUST), Wuhan, 430074 People’s Republic of China; 3 Institute of Materials Science and Devices, School of Materials Science and Engineering, Suzhou University of Science and Technology, Suzhou, 2150009 People’s Republic of China; 4 Guangxi Key Laboratory of Low Carbon Energy Materials, College of Chemistry and Pharmaceutical Sciences, Guangxi Normal University, Guilin, 541004 People’s Republic of China; 5 State Key Laboratory of Electroanalytical Chemistry, Changchun Institute of Applied Chemistry, Chinese Academy of Sciences, Changchun, 130022 People’s Republic of China; 6 University of Science and Technology of China, Hefei, 230026 People’s Republic of China

**Keywords:** Crystal facet engineering, Anisotropy, Oxygen evolution reaction, Hydrogen evolution reaction, Theoretical simulations

## Abstract

- The crystal facets featured with facet-dependent physical and chemical properties can exhibit varied electrocatalytic activity toward hydrogen evolution reaction (HER) and oxygen evolution reaction (OER) attributed to their anisotropy.
- The highly active exposed crystal facets enable increased mass activity of active sites, lower reaction energy barriers, and enhanced catalytic reaction rates for HER and OER.
- The formation mechanism and control strategy of the crystal facet, significant contributions as well as challenges and perspectives of facet-engineered catalysts for HER and OER are provided.

## Abstract (highlights)

The electrocatalytic water splitting technology can generate high-purity hydrogen without emitting carbon dioxide, which is in favor of relieving environmental pollution and energy crisis and achieving carbon neutrality. Electrocatalysts can effectively reduce the reaction energy barrier and increase the reaction efficiency. Facet engineering is considered as a promising strategy in controlling the ratio of desired crystal planes on the surface. Owing to the anisotropy, crystal planes with different orientations usually feature facet-dependent physical and chemical properties, leading to differences in the adsorption energies of oxygen or hydrogen intermediates, and thus exhibit varied electrocatalytic activity toward hydrogen evolution reaction (HER) and oxygen evolution reaction (OER). In this review, a brief introduction of the basic concepts, fundamental understanding of the reaction mechanisms as well as key evaluating parameters for both HER and OER are provided. The formation mechanisms of the crystal facets are comprehensively overviewed aiming to give scientific theory guides to realize dominant crystal planes. Subsequently, three strategies of selective capping agent, selective etching agent, and coordination modulation to tune crystal planes are comprehensively summarized. Then, we present an overview of significant contributions of facet-engineered catalysts toward HER, OER, and overall water splitting. In particular, we highlight that density functional theory calculations play an indispensable role in unveiling the structure–activity correlation between the crystal plane and catalytic activity. Finally, the remaining challenges in facet-engineered catalysts for HER and OER are provided and future prospects for designing advanced facet-engineered electrocatalysts are discussed.

## Graphical abstract

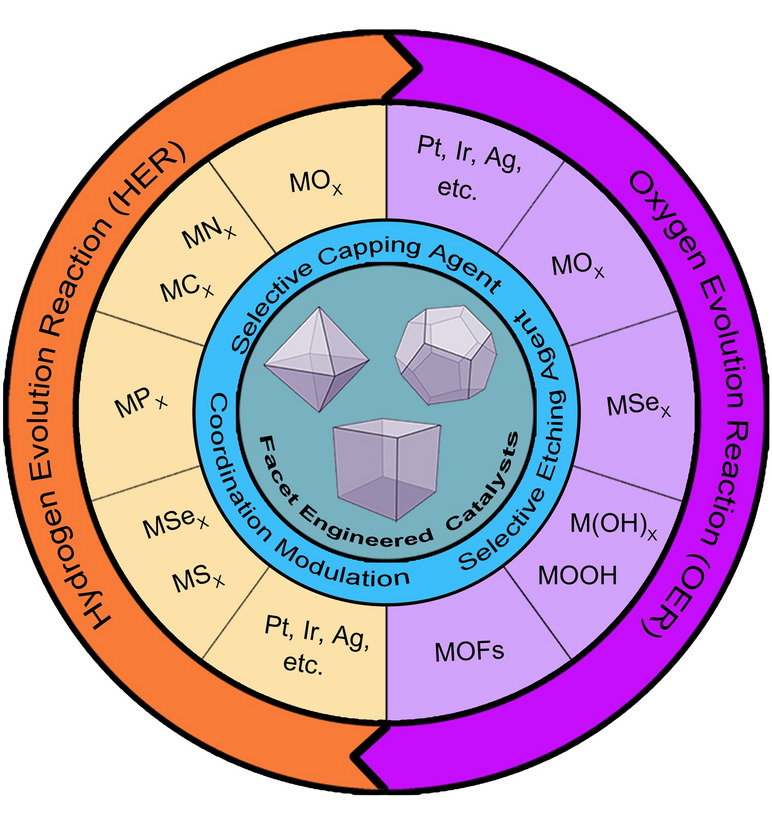

## Introduction

Due to excessive consumption of conventional fossil fuels, global energy crisis and environmental issues are arisen [1–5]. In order to achieve the future low-carbon energy economy, the exploration and utilization of green and sustainable energy sources is crucial and highly urgent. Hydrogen as the most promising energy carriers has gained increasing attention by virtue of its high energy storage capacity, carbon-free nature, and renewable nature [6–10]. Till date, hydrogen production can be realized via natural gas, other fossil fuels, and water electrolysis [1, 11]. Among these approaches, environmentally friendly electrochemical water splitting produces high-purity hydrogen, which is an efficient method to realize carbon neutrality [12–16]. However, the OER involves multiple electron transfer steps and oxygen–oxygen bond formation process, which is regarded as the main bottleneck of hydrogen production from water splitting [7, 17–21]. Furthermore, as a strongly uphill reaction, electrocatalytic splitting of water into hydrogen and oxygen typically requires a higher voltage of 1.8–2.0 V to proceed compared to the theoretical limit of 1.23 V [22, 23]. The electrocatalysts can effectively improve the sluggish kinetics and reduce the overpotentials (*η*) of HER and OER [24, 25]. Therefore, the elaborate design of HER and OER electrocatalysts for achieving high-efficiency hydrogen production and carbon neutrality is of considerable interest and great significance.

In recent decades, noble metals (Pt, Ir, Ag, etc.), transition metal oxides/phosphides/chalcogenides, metal–organic frameworks (MOFs), and their derivatives have been developed as high-performance electrocatalysts for HER and OER [19, 24, 26–49]. In order to enhance the performance of electrocatalysts, state-of-the-art strategies to tune their electronic structures and further adjust adsorption energies of oxygen or hydrogen intermediates are explored including interface engineering, doping engineering as well as defect engineering [50–69]. However, it has been overlooked that some electrocatalysts possess well-defined crystal planes with special atomic arrangements and atomic coordination environments, which might exert a positive and profound impact on electrocatalytic performance [70, 71]. Owing to the anisotropy of the crystal planes, some electrocatalysts featured with multiple crystal planes usually exhibit facet-dependent physical and chemical properties including geometric structures, surface electronic structures, surface built-in electric fields, and redox active sites, which causes differences in the adsorption energies of oxygen or hydrogen intermediates, thus leading to different electrochemical activity and selectivity toward HER or OER [30, 72–77]. Remarkably, the X-ray absorption spectroscopy (XAS) is capable of investigating the chemical states and atomic structures of metal ions in facet-engineered catalysts [78]. For instance, local structures of Ir atoms in 2D ultrathin {001}-faceted SrIrO_3_ perovskite were analyzed through the XAS. The XAS results confirmed that electron density around Ir atoms is richer and the Ir–O coordination number is 5.8 close to an ideal coordination of 6 [77]. The XAS is conducted to investigate Co oxidation states, electron occupancies, and coordination environments of PrBaCo_2_O_6_ films with the {100} facets. The XAS results indicated that PrBaCo_2_O_6_ films with the {100} facets presented more high-spin Co^3+^ states, which can tailor their electronic structure [79]. Besides, the highly active exposed facet should have more reactive active sites, which greatly improves the mass activity of active sites [80–82]. Thus, selective exposure of dominant crystal planes has been regarded as the effective strategy for achieving increased mass activity of active sites, decreasing reaction energy barriers and increasing catalytic reaction rates [83–86]. Nevertheless, it is still challenging to tune catalyst morphology to realize highly active exposed dominant crystal planes without changing the composition.

**Fig. 1:**
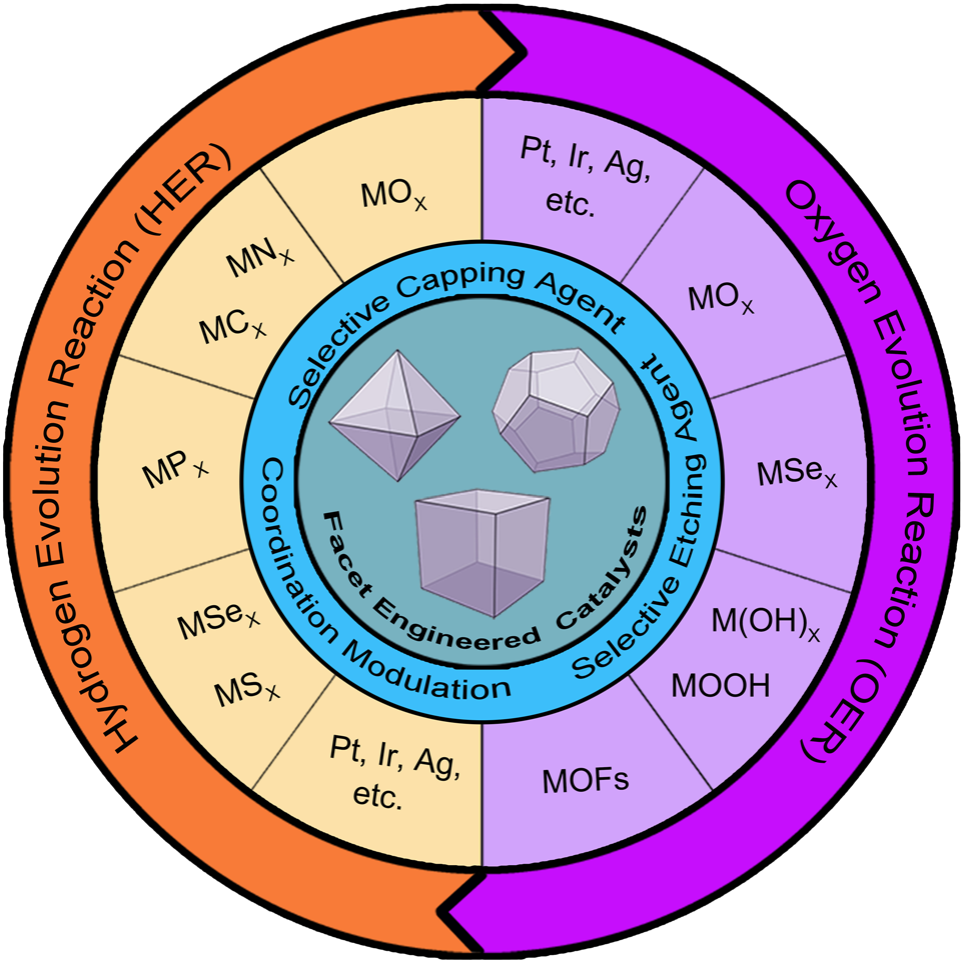
Summaries of crystal facet control strategies and facet-engineered catalysts for electrocatalysis of HER and OER. M represents transition metals, e.g., Fe, Co, Ni, Cu

The crystal morphology can roughly reflect the categories and proportions of main crystal planes [87]. In addition, different crystal planes show different surface energy, resulting in different selective toward HER or OER [30, 82, 88–91]. For example, the high-index facets (edges, steps, and kinks) featured with a high ratio of low-coordinated atoms usually exhibited a higher surface energy than low-index facets, indicating favorable surface atomic structures and hence enhanced electrocatalytic activity for the high-index facets [30, 87, 92–98]. Therefore, optimizing crystal morphology to expose highly active facets with high surface energy can effectively ameliorate electrocatalytic activity. The surface energy across the crystal surface would tend to minimize during the crystal growth process based on Gibbs–Wulff theorem [99]. More specifically, crystal facts with high surface energy would slowly decrease or even disappear, which reduces electrocatalytic activity. Fortunately, during the growth of the crystal, some additives have emerged as effective capping agents or etching agents in controlling the crystal growth process and to obtain targeted crystal morphology aiming to preserve the highly active crystal facets [72]. Crystal facet engineering is capable of controlling and further expanding the percentage of desired crystal facets on the crystal surface. Accordingly, it is an effective strategy to tailor crystal morphology with the assistance of crystal facet engineering for designing electrocatalysts dominated by high-activity facets (Fig. 1).

With the increasing demand for high-efficiency catalysts, the last decades have witnessed the boom of facet-engineered catalysts for electrochemical hydrogen and oxygen evolution. However, a comprehensive summary in facet-engineered electrocatalysts toward the HER and OER is still lacking. Herein, in this review, we provide a systematic summary of facet engineering for HER, OER, and overall water splitting (OWS) (Fig. 1). First, this review starts from the basic concepts, fundamental mechanisms, and evaluation parameters of HER and OER. Meanwhile, the formation mechanism of the crystal facet is highlighted. Then, various strategies of selective capping agent, selective etching agent, and coordination modulation to tailor crystal facets are summarized. Next, a comprehensive overview of significant contributions of facet-engineered catalysts for HER, OER, and OWS is given. Importantly, we point out that the DFT calculations play a fundamental role in accounting for the structure–activity correlation between the crystal plane and catalytic activity. Finally, the challenges and perspectives in facet-engineered catalysts for HER and OER are proposed from our point of view.

## Fundamentals of OER and HER

### HER Mechanisms

**Fig. 2:**
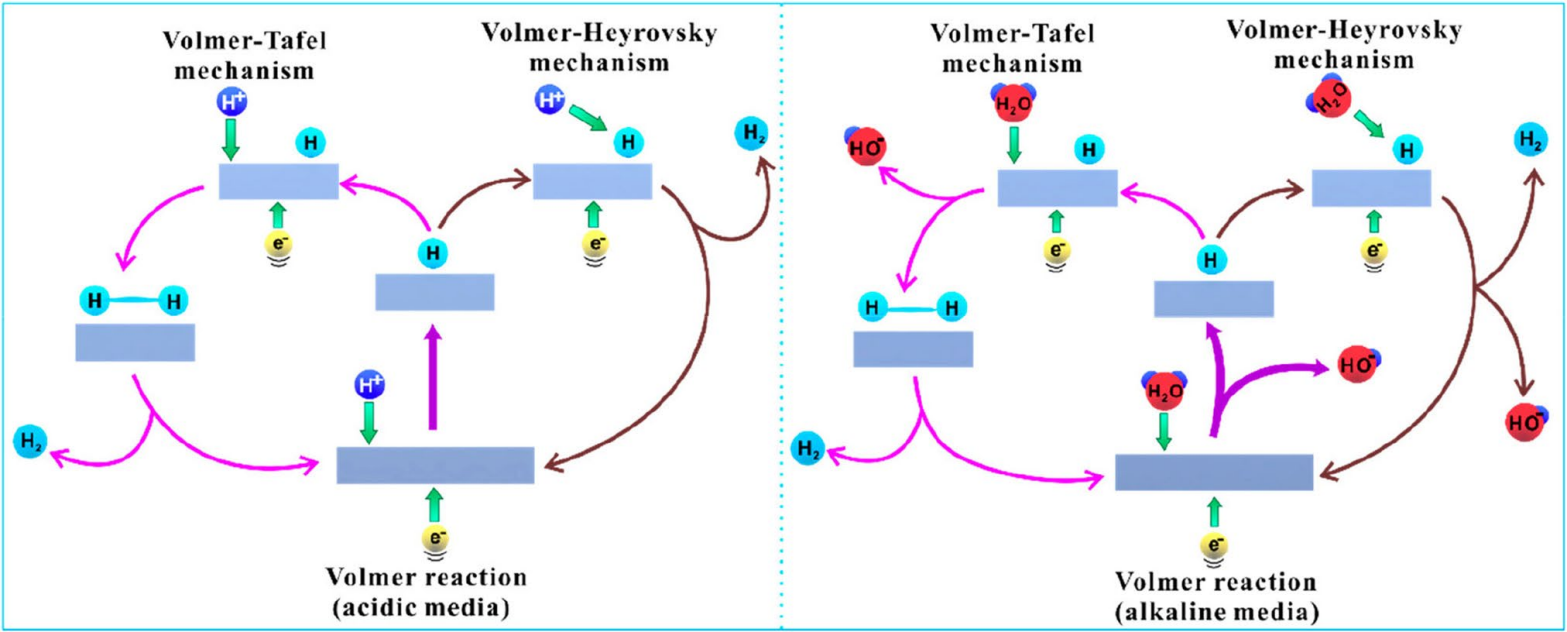
Schematic diagram of HER mechanisms in acidic media (left) and in alkaline media (right). Reproduced with permission [3]. Copyright 2020, American Chemical Society

Hydrogen production from electrochemical water splitting involves two different reaction pathways including the reduction of protons in the solution via the Volmer step and generation of hydrogen through electrochemical (the Heyrovsky step) or chemical (Tafel step) route (Fig. 2) [3, 28, 100–103]. The first step of HER is the Volmer reaction, wherein an electron (e^−^) transfers to the electrode can react with the H^+^ or H_2_O in acidic or alkaline solution on a void active site of the catalyst to produce the adsorbed hydrogen atoms (H_ads_) (Eq. (1) or (2)). Subsequently, the second step is associated with desorption and accumulation of the H_ads_ for H_2_ formation [104]. This process could be proceeded either via the Tafel step or the Heyrovsky step (Eqs. (3), (4), or (5)) [42, 105–108]. A low occupancy of H_ads_ on the catalyst surface allows it to transfer an electron and react with H^+^ in the solution to produce H_2_. This process is a Heyrovsky reaction [104]. The formation of H_2_ is caused by the bonding between adjacent H_ads_ when their occupancy is high on the catalyst surface. This process is a Tafel reaction [36]. The difference of HER mechanisms in acidic media and alkaline media lies in the proton source [3]. In acidic media, hydronium ions (H_3_O^+^) are the proton source, while in alkaline media, the proton source is derived from water molecules.

*Volmer Step*

1 $${\text{H}}^{+} + {\text{e}}^{ - } \to {\rm H}_{\rm ads} \left( {\rm acidic}\;{\rm media} \right)$$

2 $${\text{H}}_{2} {\text{O}} + {\text{e}}^{ - } \to {\text{OH}}^{ - } + {\text{H}}_{{{\text{ads}}}} \left( {{\text{alkaline}}\;{\text{media}}} \right)$$

*Heyrovsky Step*

3 $${\text{H}}^{ + } + {\text{e}}^{ - } + {\rm H}_{\rm ads} \to {\text{H}}_{2} \left({\rm acidic}\;{\rm media} \right)$$

4 $${\text{H}}_{2} {\text{O}} + {\text{e}}^{ - } + {\rm H}_{\rm ads} \to {\text{OH}}^{ - } + {\text{H}}_{2} \left( {\rm alkaline}\;{\rm media} \right)$$

5 $${\text{Tafel}}\;{\text{step}}:2{\text{H}}_{\rm ads} \to {\text{H}}_{2}$$

The Tafel slope demonstrates the potential difference required to change the current density by order of magnitudes, which is beneficial to discern the mechanism of the HER process [3, 109, 110]. In case the proton discharge reaction is rapid and H_2_ formed via a rate-determining step (RDS) of chemical desorption, a Tafel slope of 29 mV dec^−1^ is expected according to the formula (*b* = 2.3*RT*/2*F*) at 25 °C. In case the proton discharge reaction is rapid and H_2_ is produced through the rate-limiting step of electrochemical desorption, a Heyrovsky slope of 38 mV dec^−1^ is deduced from the formula (*b* = 4.6*RT*/3*F*) at 25 °C. In case the proton discharge reaction is slow, a large slope of 116 mV dec^−1^ is acquired from the formula (*b* = 4.6*RT/F*) at 25 °C.

### OER Mechanisms

**Fig. 3:**
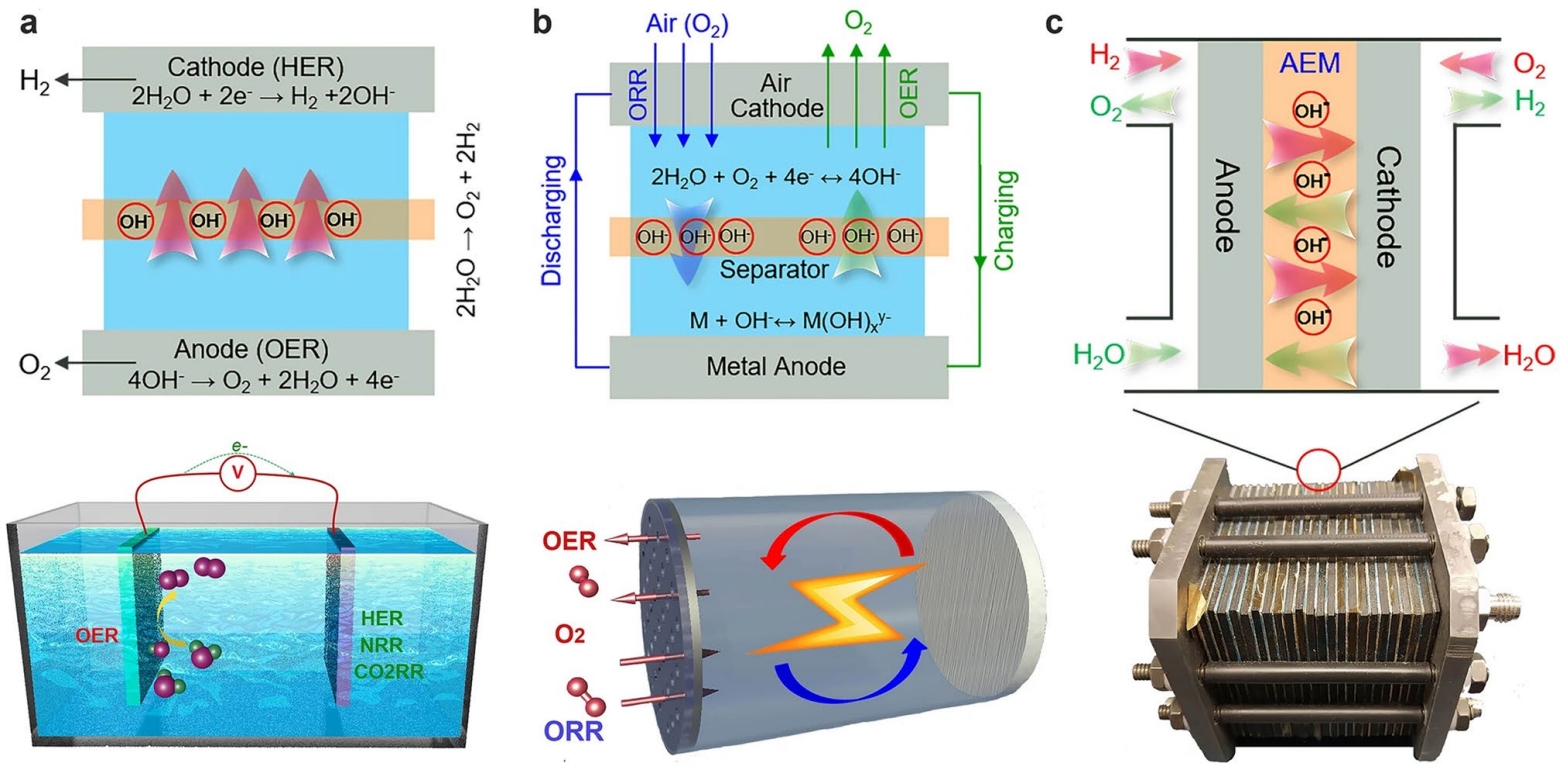
The OER involved energy conversion and storage devices: **a** small molecule electrolyzers, **b** metal–air batteries, **c** regenerative fuel cells. Reproduced with permission [114]. Copyright 2022, Springer Nature

The development and design of high-performance energy conversion and storage systems, including small molecule (water, carbon dioxide and nitrogen) electrolyzers, metal–air batteries, and regenerative fuel cells, are of essential importance for realizing carbon neutrality [111–113]. As shown in Fig. 3, the OER has been regarded as a key reaction process among these electrochemical devices, which can build a bridge between renewable electricity and chemical fuels [114]. For example, the OER can proceed at the anode during the electrolysis of small molecules (Fig. 3a), while the OER occurs on the cathode in metal–air battery (Fig. 3b). A regenerative fuel cell requires participation of powerful oxygen electrolysis to produce high electrical energy (Fig. 3c). However, sluggish kinetics of OER reduces the electrochemical efficiency of these electrochemical devices. The design and exploration of high-performance OER catalysts for improving the efficiency of energy conversion and storage systems are a key step of carbon neutrality. Therefore, in the following, a brief summary of OER mechanisms is presented intending to rational design OER electrocatalysts with high catalytic activity.

#### Conventional Adsorbate Evolution Mechanism

**Fig. 4:**
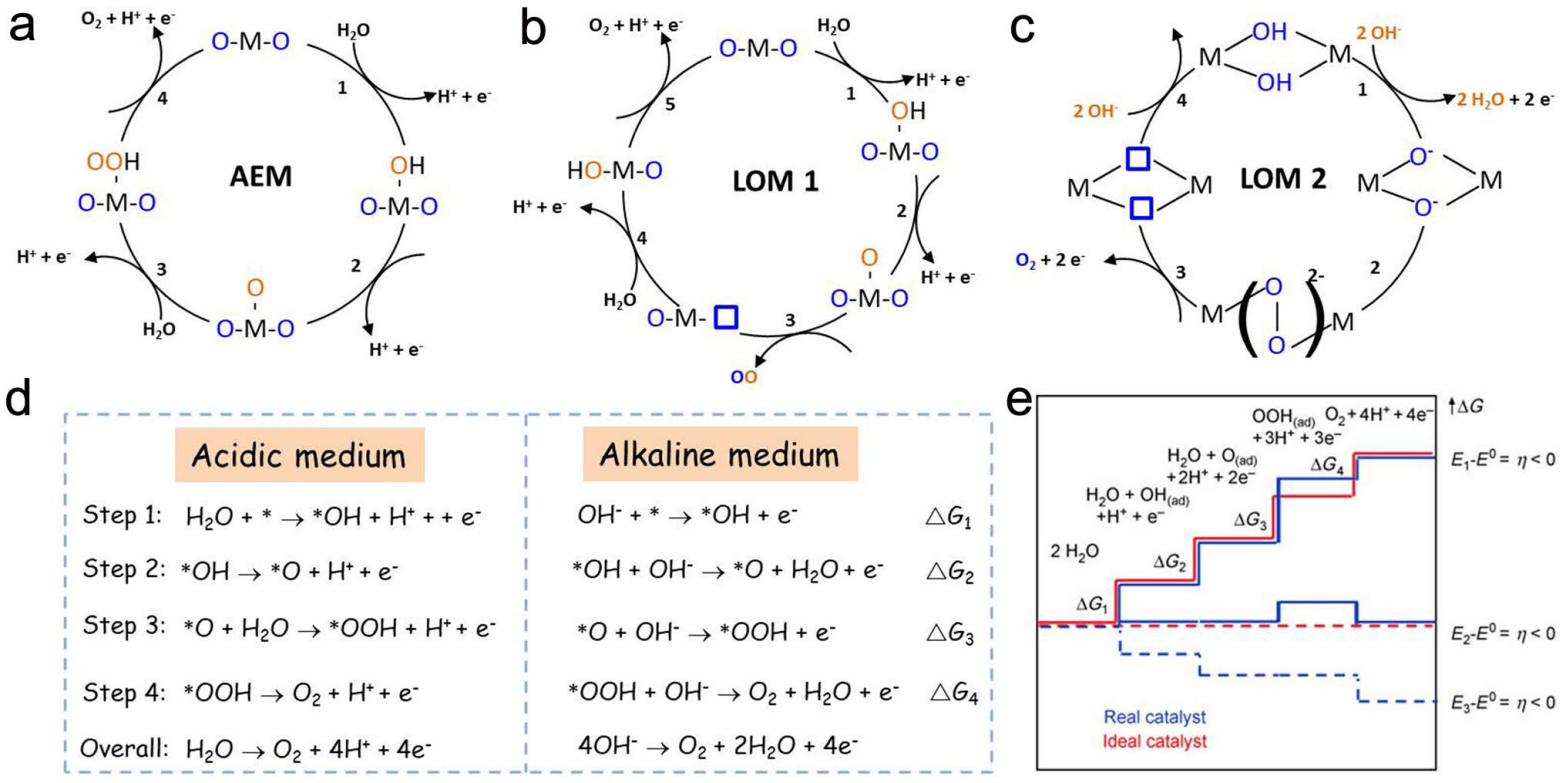
**a** AEM for OER. **b** LOM 1 for OER with one oxygen atom originated from the catalyst to form oxygen molecular. **c** LOM 2 for OER with both oxygen atoms of oxygen molecular derived from the catalyst. **d** Schematic diagram of AEMs for OER in acidic and alkaline electrolyte. **e** OER Gibbs free energy diagram versus reaction pathways for reactive species and intermediates. Red and blue lines represent ideal and real reaction pathways, respectively. Reproduced with permission [125]. Copyright 2010, Wiley-VCH Verlag GmbH & Co

Mechanistically, oxygen formation occurs on the surface of the anode electrode in two possible reaction pathways: one is the conventional adsorbate evolution mechanism (AEM) and another is the lattice oxygen mechanism (LOM) as depicted in Fig. 4a-c [115–121]. Generally, the OER process of AEM in acidic medium or alkaline medium undergoes four concerted proton–electron transfer steps with multiple oxygenated intermediates (*OH, *O and *OOH) [106, 122, 123]. Taking the alkaline OER process of AEM as an example (Fig. 4d), hydroxide anions firstly absorb on the active site (*) through one-electron oxidation process, producing an *OH. Subsequently, *OH is coupled to hydroxide anions forming *O. Then, O* combines with another OH to generate *OOH. O_2_ is produced through the deprotonation of *OOH with the restoration of initial activity. In theory, the energy barrier of OER might be originated from each of the four steps. The step with the largest positive value among Δ*G*_1_ ~ Δ*G*_4_ is identified as the RDS and determines the theoretical *η* of OER [124]. As displayed in Fig. 4e, for an ideal catalyst, all four reaction steps would have a reaction free energy of 1.23 eV at U = 0 [125]. However, catalysts cannot achieve this ideal situation in practice because oxygenated intermediates have linearly related adsorption energies [126]. Particularly, the O atom from both *OH and *OOH binds with the surface of catalyst by a single bond [126]. Thus, a linear relationship exists between *OH and *OOH, where the slope is approximately 1 and the intercept is 3.2 eV, which only affects the oxygenated intermediates' interactions with the catalyst surface. Interestingly, ΔG_*OOH_ can be deduced from ΔG_*OH_, resulting in a lower limit for *η* of OER. Since two proton–electron transfer steps of an ideal catalyst possess the energy separation of 2.46 (2 × 1.23) eV, the lowest theoretical *η* of 0.37 eV (i.e., [3.2–2.46 eV]/2) is acquired [114, 126, 127]. In consideration of a constant adsorption energy difference of 3.2 eV between ∆G_*OOH_ and ∆G_*OH_, the OER overpotential is determined by *O adsorption energy. In other words, the second or third reaction step might be the RDS for OER [126].

#### Lattice Oxygen Mechanism

The traditional AEM followed by the scaling relationship between *OOH and *OH cannot surpass the limitation of minimal overpotential of 0.37 V [116, 128–130]. Fortunately, the emerged LOM can offer a unique reaction pathway for the occurrence of O–O bond coupling, wherein it would neither fully undergo reaction pathways of the AEM and nor restricted by the scaling relationship of the oxygenated intermediates [27, 131, 132]. As shown in Fig. 4b, c, there are two types of LOM reaction pathways, depending on the number of oxygen atom derived from catalysts [50, 128, 133]. The first two steps of LOM 1 and LOM 2 are consistent with the AEM for OER. In the third step of LOM 1, the *O species can couple with the lattice oxygen in the catalyst to yield an O_2_ molecule, which generates oxygen vacancy in the catalyst simultaneously. Next, the oxygen vacancy is refilled by a water molecule, producing *OH on the M site of M–O. Finally, H is released to yield refreshed M active sites [132]. For LOM 2, two *OH on the M firstly go through deprotonation intending to producing two metal-oxo species. Subsequently, an O–O bond is formed by coupling two oxo species. Finally, oxygen gas is evolved through exposing two oxygen vacancies occupied by OH [128, 134].

### Key Evaluating Parameters of the HER and OER

#### Overpotential

Due to the kinetic energy barrier, electrocatalytic OER or HER can proceed through the additional potential to drive oxygen or hydrogen production. The additional potential is named overpotential (*η*), which is obtained from the potential difference between the experimental value and the thermodynamic equilibrium value [104]. *η* is regarded as one of the dominating factors to evaluate the electrocatalytic performance [135]. *η* at a geometric current density of 10 mA cm^−2^ originated from the potential corresponding to photoelectrocatalytic H_2_O splitting efficiency of 10% is widely accepted to evaluate the HER and OER performances of the electrocatalysts [136, 137]. Generally, an electrocatalyst with high catalytic activity would exhibit lower overpotential.

#### Tafel Slope

The Tafel slope (*b*) depicts the reaction kinetics of the electrochemical reaction, which can offer valuable and insightful information toward reaction mechanisms, especially for elucidating the RDS [138]. The Tafel slope is calculated from the linear portion of the Tafel plot. The Tafel equation is expressed as *η* = *a* + *b*log (*j/j*_*0*_*)*, where *a* represents the constant determined by temperature, electrode properties, and electrolyte [11], *j* is the total current density at a given *η*, and *j*_*0*_* is* the exchange current density. The Tafel value is associated with the reaction mechanisms, especially for the HER [3, 138]. *j*_*0*_ reveals the intrinsic activity of a specific electrocatalyst under equilibrium conditions [109]. Generally speaking, an electrocatalyst with high catalytic activity would exhibit a small Tafel slop (*b*) and a high *j*_*0*_.

#### Stability

Stability performance is regarded as another crucial parameter in evaluating catalysts for practical applications. The first approach to evaluate the stability is comparing the change between the linear sweep voltammetry (LSV) curve before and after a certain number of cyclic voltammetric scans [11]. The second method of stability test is conducted via monitoring the current density (potential) variation with time at a fixed potential (current density) [139]. The third method (denoted as multi-step chronopotentiometric test) to judge the stability is to monitor the changes of potential (corresponding to the same current density) after running at varied current densities within multiple cycles. Generally, a smaller change at the same current density indicates better stability toward HER and OER.

#### Turnover Frequency

Turnover frequency (TOF) is another vital parameter for evaluating the intrinsic activity of an electrocatalyst [139]. The TOF value can be deduced from the following equation: TOF = (*j* × *A*) × (n × F × *m*)^−1^, where A represents the area of working electrode, n refers to the electron transfer number at a specific *η*, F stands for the Faraday constant, and m is the mole number of metal on the surface of catalyst. Thus, an electrocatalyst with a lager TOF value demonstrates the higher intrinsic activity.

#### Faradaic Efficiency

Faradaic efficiency (FE) depicts the electron conversion efficiency for hydrogen or oxygen production. The FE value of HER or OER is the ratio of experimental value to theoretical value of H_2_ or O_2_ production [124, 140]. The practical amount of H_2_ or O_2_ production is detected through gas chromatography (GC) or the conventional water**–**gas displacement method, and the theoretically yield of hydrogen or oxygen is obtained via the chronoamperometric or chronopotentiometric analysis [141].

#### Electrochemically Active Surface Area

Electrochemically active surface area (ECSA) can reflect the electrocatalytic performances of HER/OER catalysts through evaluating the specific surface area and the number of active sites [19]. The ECSA is positively correlated to the double-layer capacitor (*C*_dl_), which is usually obtained by analyzing CV curves of varied scanning rates in the non-faradaic region [130]. The large *C*_dl_ value for the catalyst indicates abundant catalytically active sites and thus presents enhanced electrocatalytic performances during the HER/OER process.

## Formation Mechanism of the Crystal Facet

**Fig. 5:**
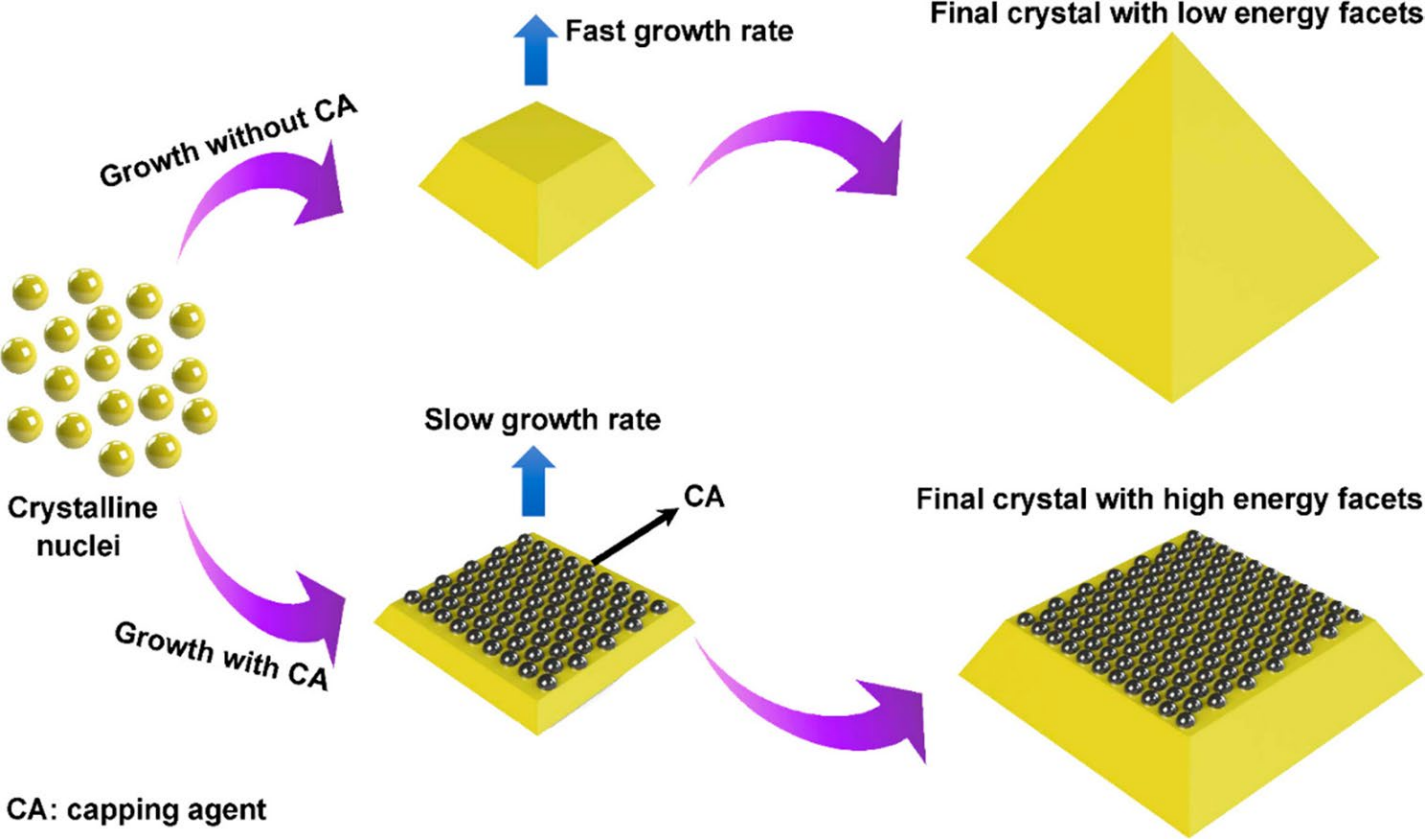
Schematic diagram of organic or inorganic additive used for crystal facet engineering. Reproduced with permission [72]. Copyright 2019, American Chemical Society

The formation of the crystal involves two steps: nucleation and growth. When the saturation of a solution exceeds a critical value, crystal nuclei are produced [142, 143]. When the size of the crystal nucleus is larger than the critical size, its Gibbs free energy changes more than the surface energy and the crystal nucleus gradually grows to form a crystal. Remarkably, the crystal eventually evolves into a polyhedral shape composed of planes with different orientations during its growth process. These planes with different orientations are called crystal facets, which can be generally expressed by a set of Miller indices {*hkl*} [87]. The generation of the crystal facet needs to follow the Gibbs–Wulff theorem during the growth of the crystals [99]. As presented in Fig. 5, the crystal facets with higher surface energies allow fast growth rates and usually occupy a small part of the surface or even disappear as the crystal grows, while the crystal planes with lower surface energies grow slowly and take up larger proportions of the surface, which constitutes the main part of the entire crystal facet [72]. Therefore, the surface energy of the crystal facet can be tailored by using organic or inorganic additives during the crystal growth process, thus achieving shape-controlled synthesis of crystals with desired crystal facets. Nowadays, with the progress of science and technology, the crystal facets of materials can be characterized and further identified through the collaborative combination of various advanced instruments including powder X-ray diffractometer (XRD), high-resolution transmission electron microscope (HR-TEM), high-angle annular dark-field scanning transmission electron microscope (HAADF-STEM), etc. Specifically, in the XRD test, a specific angle corresponds to a specific diffraction peak, which can identify theoretical crystal facets and provide the theoretical interplanar distance. HR-TEM and HAADF-STEM instruments can measure interplanar distances of crystal facets at ultra-high multiples, which is mutually verified with XRD results.

## Various Strategies of Crystal Plane Control

### Capping Agent with Crystal Plane Selectivity

**Fig. 6:**
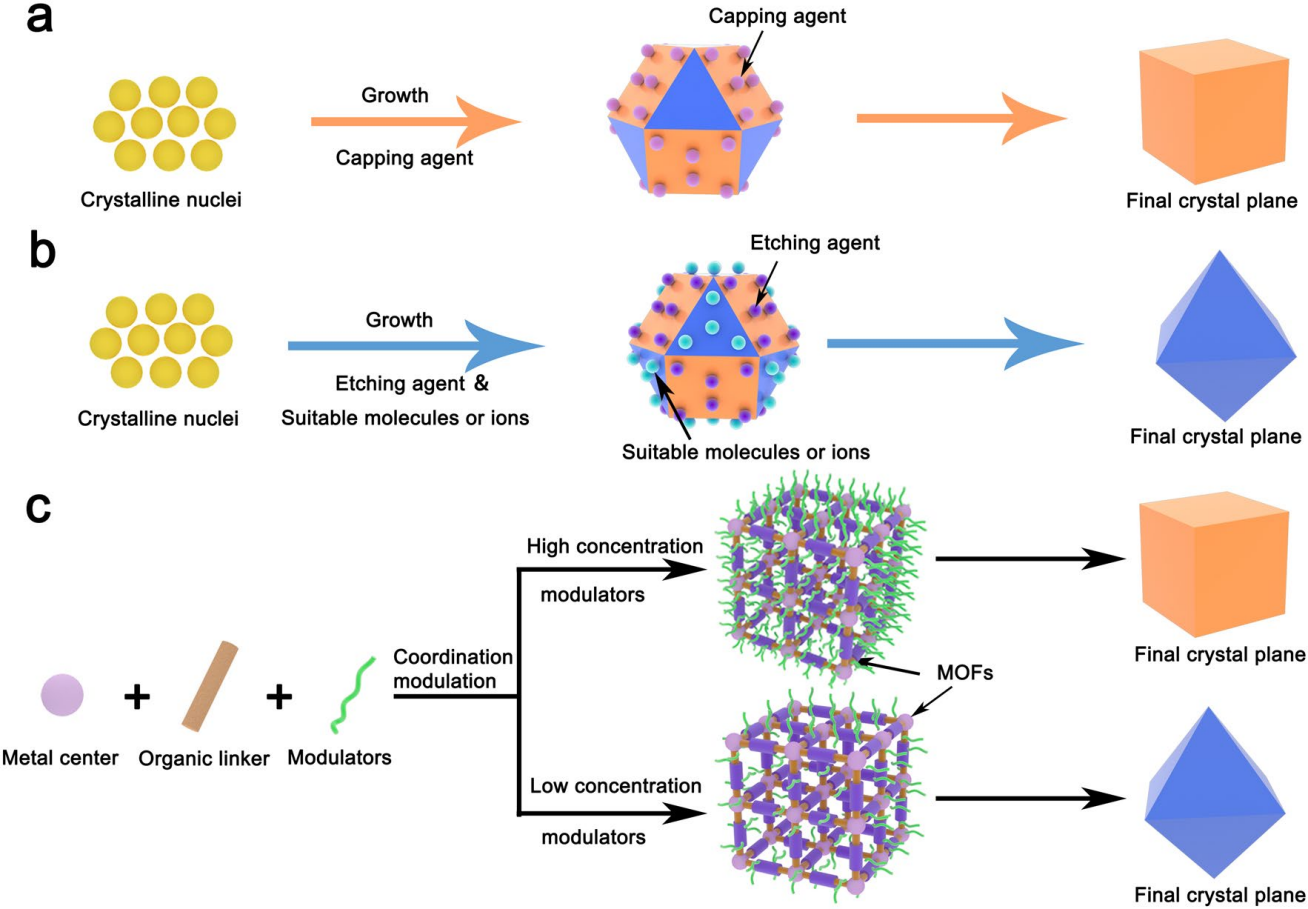
Schematic illustration of crystal plane control using **a** selective capping agents, **b** selective etching agents, and **c** modulators

As shown in Fig. 6a, capping agent can selectively attach on some specific crystal planes with higher surface energies and lower the surface energies to slow down the growth rate of the adsorbed planes, therefore expanding the percentage of the adsorbed planes (exposed planes) on the entire crystal surface [144]. Thus, the proportion of different crystal planes on the entire surface can be determined through controlling the amount of capping agent added. For instance, Yang and coauthors revealed that the anatase TiO_2_ is exposed with {001} facets by increasing the concentration of fluoride ions (as the capping agent) to adjust the order of surface energy [145]. As result, the percentage of exposed {001} facets reached 47% while only 4% was obtained without fluoride ions. Subsequently, inspired by the above, anatase TiO_2_ crystals with the exposure of 60%, 64%, and 89% {001} facets are produced by further optimizing the experiments [146–148]. Zhang et al. [149] realized the evolution of Cu_2_O nanocrystals from nanocubes to truncated nanocubes, nanocubooctahedrons, truncated nanooctahedrons, and finally nanooctahedrons through changing the amount of polyvinylpyrrolidone (PVP). Cu_2_O exposed different facets during the evolution. The Cu_2_O cubes with the exposure of {100} facets can be obtained without the addition of PVP, while with the addition of PVP increased, the Cu_2_O cubes eventually transform into the Cu_2_O octahedrons with the exposed {111} facets. Zeng et al. [144] found that citrate and PVP can selectively attach to surfaces of Ag nanoparticles to decelerate the grow rate, resulting in the generation of Ag octahedrons with {111} facets and Ag nanocubes/nanobars with {100} facets, respectively.

### Etching Agent with Crystal Plane Selectivity

As mentioned above, capping agent strategy is adopted to control crystal planes during crystal formation. Yet, etching agent strategy is used to tailor crystal planes after crystal formation. Intriguingly, two strategies have a similar effect on crystal plane control: The basic principle is all originated from the selective effect between the added reagent and the crystal plane. Etching agent strategy is a top-down route for the preparation of targeted planes. The top-down approach involves selecting certain molecules or ions that can selectively attach to the targeted planes, avoiding them being damaged from etching agent (Fig. 6b). In theory, targeted planes will be preserved and other undesirable planes will be dissolved by the etching agent. However, surface recrystallization phenomena may occur on the mother crystal facets during the etching process [99]. Besides, different planes may demonstrate diverse anticorrosion property to a specific etching reagent. Hence, it is important to choose a proper etching agent for the construction of desired facets. For instance, Pal et al. [150] proposed a simple, facile, and surfactant-free approach to prepare five types of Cu_2_O nanoparticles with different shapes. The morphological evolution of Cu_2_O nanoparticles can be achieved attributed to the etching agents (NaOH solution, triethylamine (TEA), and oxalic acid solution). After the etching process, NaOH-etched, TEA-etched, and oxalic acid-etched products of octahedral Cu_2_O nanoparticles exhibited the exposed {110} facets, {111} facets, and {110} as well as {200} facets, respectively. Tsai et al. [151] achieved precise etching {110} facets of Cu_2_O nanocrystals by adding HCl during the growth of Cu_2_O nanocrystals. Liu et al. [152] found that {001} facets of anatase TiO_2_ can be selectively etched by HF and corresponding {101} facets were preserved. During the etching process, the {001} facets were slightly etched or fully etched by tuning the concentration of HF.

### Coordination Modulation

The tunable of crystal planes is feasible using modulators via the coordination modulation method. Notably, the coordination modulation method to control crystal planes is currently suitable for MOFs. As displayed in Fig. 6c, the role of the coordination modulator is to control the kinetics of diverse processes (e.g., nucleation) and induce the crystal orientation growth in a specific axis, thus leading to the generation of the crystal with specific and desirable facets [153–155]. For instance, Umemura et al. [154] prepared a porous coordination framework of [Cu_3_(btc)_2_]_n_ (btc is benzene-1,3,5-tricarboxylate) assembled with btc as the organic ligand and copper(II) nitrate trihydrate as the metal source. The morphological evolution of [Cu_3_(btc)_2_]_n_ from octahedron and cuboctahedron to cube was achieved by increasing the concentration of lauric acid (as the modulator). When the concentration of modulator was low, [Cu_3_(btc)_2_]_n_ with exposed dominant {111} facets was obtained, whereas when the concentration of modulator was high, the resulting [Cu_3_(btc)_2_]_n_ exposed dominant {100} facets. Yang et al. reported that coordination modulation of MIL-125 crystals with truncated octahedral shape was realized by using butyric acid as the modulator. The as-prepared MIL-125 crystals consisted of two exposed {001} and eight {101} facets. Sikdar et al. [156] realized the morphological and dimensionality transition with diverse crystal facets through varying the concentration of dodecanoic acid (as a modulator). As-obtained hex-MOF1, rod-MOF1, and meso-MOF1 were dominated by {200} planes, {075} and {07–5} planes, and {030} planes, respectively.

## Facet-Engineered Catalysts for HER

### Noble Metal Catalysts

**Fig. 7:**
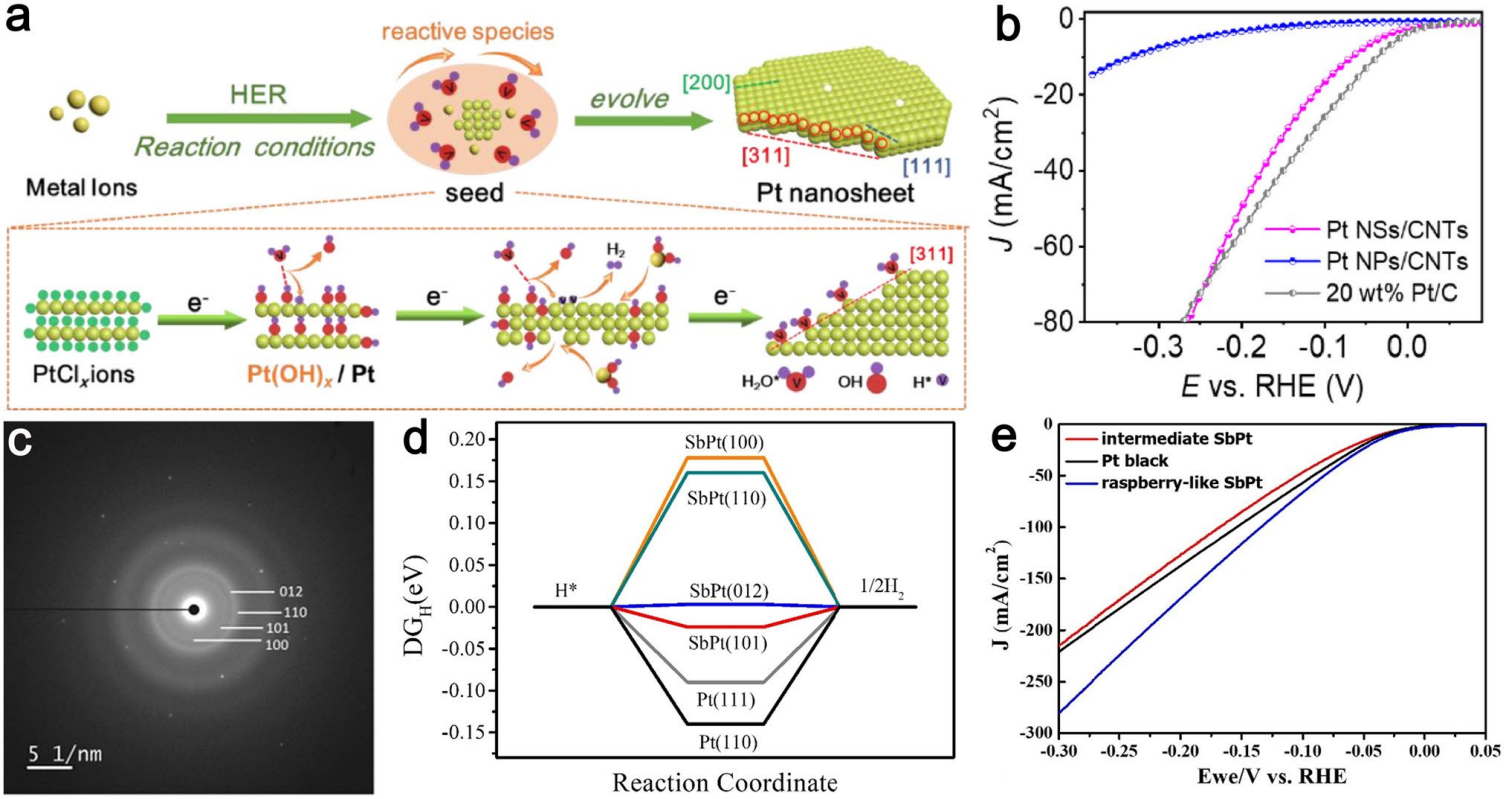
**a** Schematic diagram of the formation of Pt NSs/CNTs with the exposure of {311} and {200} facets under in situ HER conditions. **b** HER polarization curves of Pt NSs/CNTs, Pt NPs/CNTs, and 20 wt% Pt/C. Reproduced with permission [160]. Copyright 2020, Elsevier B.V. and Science Press on behalf of Science Press and Dalian Institute of Chemical Physics, Chinese Academy of Sciences. **c** Indexed SAED pattern of SbPt nanoparticles. **d** HER free energy diagram for SbPt (100), (110), (012), and (101) as well as Pt (111) and (110). **e** LSV curves of intermediate SbPt, Pt black, and raspberry-like SbPt. Reproduced with permission [162]. Copyright 2020, Elsevier Inc

Noble metal catalysts, especially Pt-based nanocatalysts with optimal hydrogen absorption energy, are considered as the promising HER catalysts [157, 158]. The proportions of various crystal facets on the Pt nanocrystal surface can be regulated by changing its morphology. For instance, the cuboctahedron of the Pt nanocrystal is mainly composed of {111} facets and {100} facets, while its cube and tetrahedral are dominated by {100} facets and {111} facets, respectively [159]. As shown in Fig. 7a, Bao and co-authors proposed an in situ electrochemical reduction strategy to synthesize Pt nanosheets anchored on carbon nanotubes (Pt NSs/CNTs) with high-index {311} facets as well as low-index {200} and {111} facets during the HER process [160]. Theoretical calculations and experimental results revealed that adsorbed H_2_O was served as capping agents to protect the {311} and {200} facets of Pt nanosheets. Thus, the remaining {200} facets performed as efficient active sites for H_2_ recombination. As displayed in Fig. 7b, Pt NSs/CNTs exhibited excellent HER activity, requiring low *η* of 36 and 175 mV to achieve current densities of 10 and 50 mA cm^−2^ in 1.0 M KOH, respectively. The corresponding Tafel slope is 44 mV dec^−1^. Sun et al. [161] fabricated Pt nanodendrites (Pt NDs) with the exposure of {111} facets at the surface of activated carbon via direct electrochemical deposition. Benefiting from the exposed numerous atoms at {111} facets and high surface area, the Pt NDs showed distinguished electrocatalytic activity toward HER with a low *η* of 27 mV at 10 mA cm^−2^, a small Tafel slope of 22.2 mV dec^−1^, and prominent stability for more than 6 h or even 5000 CV cycles of continuous electrolysis in 0.5 M H_2_SO_4_ solution. Moreover, Pt NDs also display good catalytic performance within a wide pH range, producing 30–45% more hydrogen compared to that of the commercial catalyst with the same content of Pt. Raspberry-like antimony–platinum (SbPt) nanoparticles with exposed {110}, {100}, {101}, and {012} facets (Fig. 7c) were obtained by Chan et al. [162]. According to the proposed theoretical structural model, the density functional theory (DFT) results demonstrated that SbPt nanoparticles featured different adsorption energies for {101} and {012} facets, which were smaller compared to those of the classic Pt crystal planes such as {111} facets and {110} facets (Fig. 7d). The crystal facets of SbPt nanoparticles can be moderated for HER pathway in the chemisorption of H*, resulting in improved HER catalytic activity. As a result, the raspberry-like SbPt nanoparticles with highly active {110}, {100}, {101}, and {012} facets exhibited superior HER activity with a small Tafel slope of 50.5 mV dec^−1^ and a small *η* of 81 mV to reach 10 mA cm^−2^ in 0.5 M H_2_SO_4_ solution (Fig. 7e).

Apart from Pt-based electrocatalysts, other precious metal-based materials (e.g., Ag, Pd, etc.) are also promising electrocatalytic materials for HER. For example, Kuo et al*.* [163] designed and synthesized {100} facets-dominated silver nanocubes (Ag NCs) and {111} facets-dominated silver nanooctahedra (Ag NOs) through the polyol reaction. The Ag NCs and Ag NOs possessed the light-harvesting capabilities for the plasmon-enhanced HER electrochemical system. Plasmonic Ag NOs with exposed {111} facets showed better HER catalytic performance than that of Ag NCs with exposed {100} facets in the mixture of 0.5 M H_2_SO_4_ and 1.0 M C_2_H_5_OH. The DFT calculation revealed that adsorption energy of the surfaces originated from Ag {111} facets is lower compared to that of Ag {100} facets, demonstrating that hydrogen could be easily desorbed on Ag {111} facets during the HER process. Xu et al. [164] proposed a bottom-up synthetic strategy to prepare ultrathin palladium nanosheets (Pd NSs). The key to prepare ultrathin Pd NSs with different exposed facets is to utilize the amphiphilic function of surfactants. Functional groups (carboxyl, pyridyl, quaternary ammonium, etc.) and halide counter ions of surfactants could be attached to different Pd planes, resulting in ultrathin Pd NSs with exposed {100}, {110}, and {111} facets, respectively. The surface facet-dependent HER catalytic properties of ultrathin Pd NSs were systematically explored in 0.5 M H_2_SO_4_ solution. The results evidenced that the electrocatalytic performances of three exposed facets toward HER followed the order of {111} < {110} < {100}. Pd NSs {100} displayed a *η* of 67 mV at 10 mV cm^−2^ which was much smaller than those of Pd NSs {111} (160 mV) and Pd NSs {110} (91 mV). The better HER catalytic activity of Pd NSs {100} might be attributed to the optimal balance between adsorption/desorption of H on the {100} facets of Pd NSs during HER process.

### Metal Phosphide Catalysts

Owing to the exceptionally high activity and chemical stability, metal phosphide catalysts are regarding as promising candidates to replace Pt counterparts for HER [165–167]. Yan et al. [168] adopted a facile method to synthesize edge-rich nickel phosphide nanosheet arrays on nickel foam (Ni_2_P NSs-NF). On account of abundant active edges, the Ni_2_P NSs-NF showed superior catalytic activity toward HER in both acidic and alkaline media and required a low η of 67 mV in the acidic media (0.5 M H_2_SO_4_) and 89 mV in the alkaline media (1.0 M KOH) to generate 10 mA cm^−2^ with a small Tafel slope of 57 and 82 mV dec^−1^, respectively. The Ni_2_P NSs-NF exhibited exceptionally high activity toward HER because the free energy of hydrogen adsorption (ΔG_H_) on {211} crystal planes was close to zero (− 0.03 V). In another work, {001} facets-dominated hollow Ni_2_P nanoparticles were synthesized by Popczun et al. [167]. The exposed {001} facets of Ni_2_P nanoparticles have been predicted to possess the highest catalytic performance toward HER according to the previous report [169]. As expected, the Ni_2_P nanoparticles on the glassy carbon produced 20 and 100 mA cm^−2^ at a small *η* of 130 and 180 mV, respectively, and demonstrated a low Tafel slope of 30 mV dec^−1^ in 0.5 M H_2_SO_4_. Wang et al. fabricated flower-like Ni_5_P_4_ microballs with the exposure of high-energy {001} facets. The flower-like Ni_5_P_4_ microballs featured hierarchical structure and high-energy {001} facets, which is beneficial to accelerate electron transfer and boost the inherent catalytic activity. The flower-like Ni_5_P_4_ microballs achieved outstanding HER performances with a low Tafel slope of 48 mV dec^−1^ and a small η of 35.4 mV at 10 mA cm^2^ in 0.5 M H_2_SO_4_, as well as a small Tafel slope of 56 mV dec^−1^ and a small η of 47 mV at 10 mA cm^2^ in 1.0 M KOH, respectively.

**Fig. 8:**
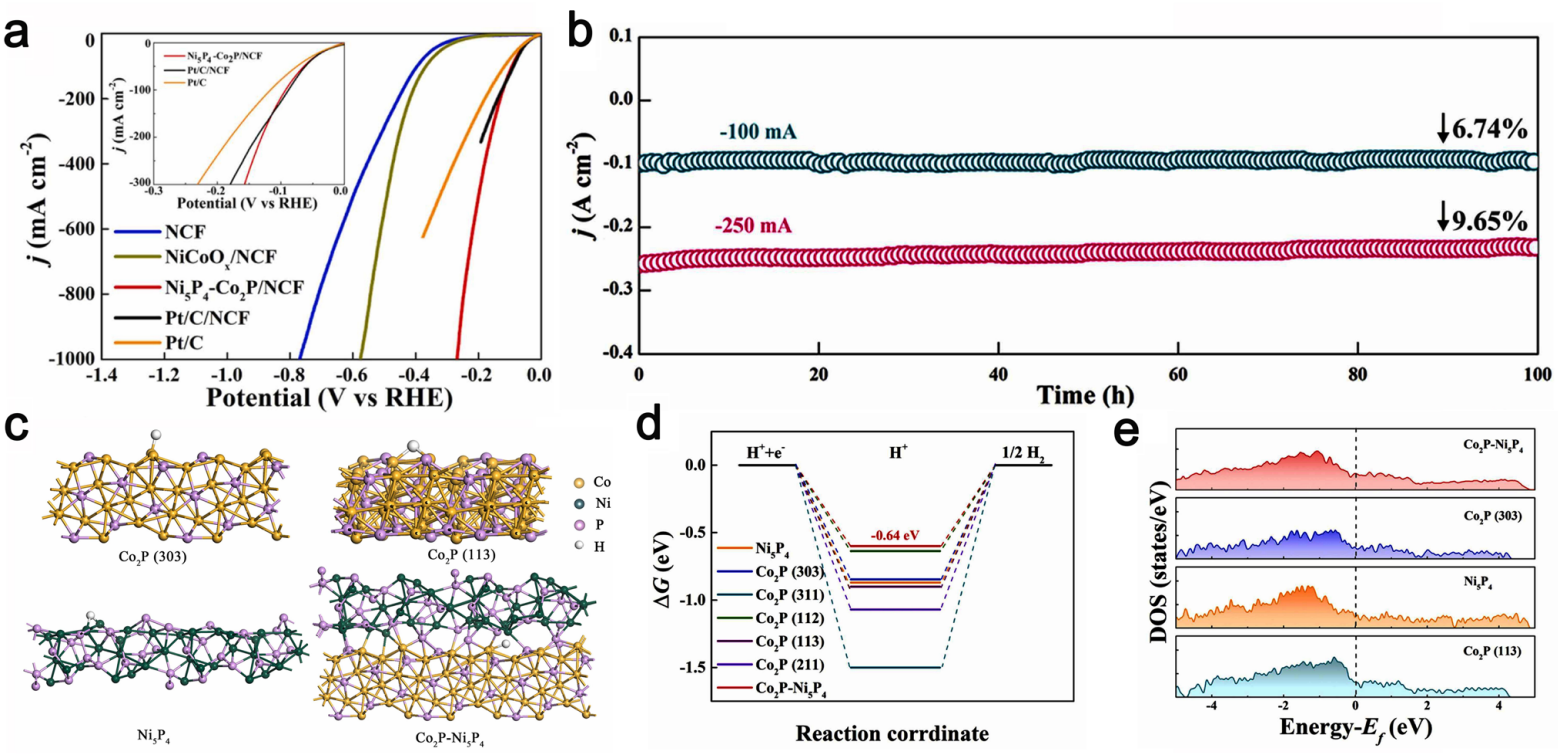
**a** HER polarization curves of Pt/C, Pt/C/NCF, NCF, NiCoO_x_/NCF, and Ni_5_P_4_-Co_2_P/NCF. **b** Chronoamperometry measurements of Ni_5_P_4_-Co_2_P/NCF maintained at *j*_100_ and *j*_250_ for 100 h. **c** Theoretical computational models of Co_2_P (303), Co_2_P (311), Co_2_P (112), Co_2_P (113), and Co_2_P (211). **d** The HER free energy diagram for Co_2_P (303), Co_2_P (311), Co_2_P (112), Co_2_P (113), Co_2_P (211), Ni_5_P_4_, and Co_2_P–Ni_5_P_4_. **e** Density of states corresponding to theoretical computational models. Reproduced with permission [170]. Copyright 2022, Elsevier Ltd

Bimetallic phosphides have been explored as the effective electrocatalysts for HER attributing to high conductivity, abundant active sites and the cooperative effects of bimetal [47, 48, 107]. For example, Ma et al. [170] constructed hetero-structured Ni–Co phosphide nanowires on the Ni–Co alloy foam (Ni_5_P_4_–Co_2_P/NCF) through a simple and versatile method. The rich heterointerfaces of the highly active {303} crystal planes between Co_2_P and Ni_5_P_4_ endowed Ni_5_P_4_–Co_2_P/NCF with the high intrinsic catalytic property toward HER, requiring a *η* of 21, 92, and 267 mV to reach 10, 100, and 1000 mA cm^−2^, respectively (Fig. 8a). The as-prepared Ni_5_P_4_–Co_2_P/NCF showed a low Tafel slope of 23 mV dec^−1^ and good stability over 100 h at 250 mA cm^−2^ in 1.0 M KOH (Fig. 8b). According to the proposed model of phosphides (Fig. 8c), the DFT calculations revealed the electronic state of the {303} crystal planes of Ni_5_P_4_–Co_2_P/NCF crossed the Fermi level (0 V) and demonstrated its metallic property beneficial to accelerate electron transfer rate (Fig. 8d). As illustrated in Fig. 8e, the Ni_5_P_4_–Co_2_P {303} model exhibited a higher electronic density around the Fermi level than those of Co_2_P {303}, Co_2_P {113}, and Ni_5_P_4_, resulting in favorable adsorption of free radicals during the HER process for the Ni_5_P_4_–Co_2_P {303} model. Yu’s [171] group synthesized the CoP/Ni_2_P hybrid catalysts via the solvothermal synthesis with post-phosphorization treatment. The CoP/Ni_2_P hybrid catalysts presented distinguished catalytic activity toward HER with a low *η* of 36, 54, and 57 mV at 10 mA cm^−2^, respectively, and a small Tafel slope of 41.2, 47.3, and 58.6 mV dec^−1^ in 0.5 M H_2_SO_4_, 1.0 M phosphate puffer solution (PBS) and 1.0 M KOH, respectively. The DFT results confirmed that the superiority of the CoP/Ni_2_P hybrid was contributed to the strong synergistic effects originated from the CoP {101} and Ni_2_P {001} planes, thus boosting the overall HER activity with an optimal ∆*G*_H_ close to 0.08 eV.

### Metal Chalcogenide Catalysts

**Fig. 9:**
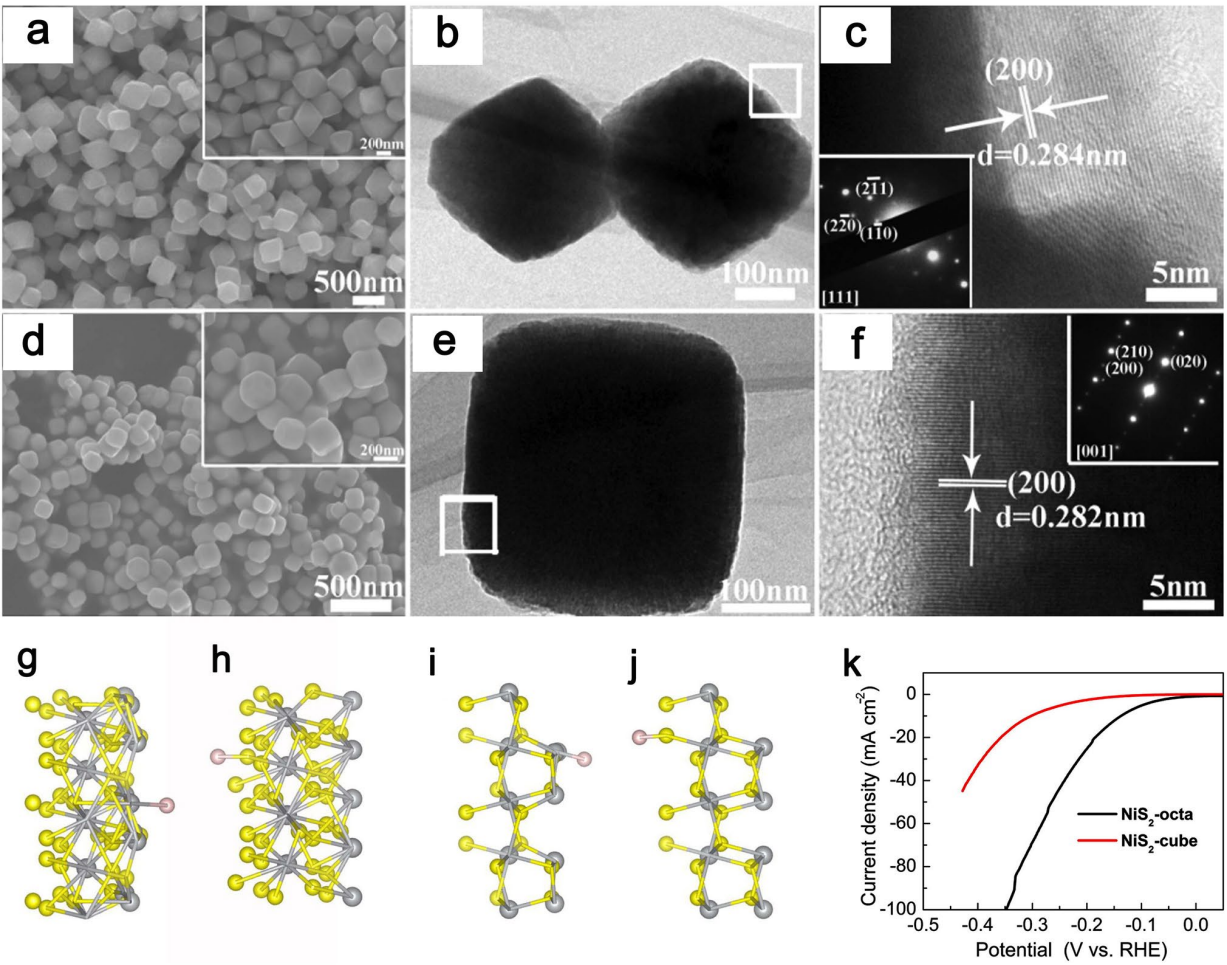
**a**–**c** SEM and TEM images of NiS_2_-octa. **d**–**f** SEM and TEM images of NiS_2_-cube. Views of the H absorbed on the **g** Ni-terminated (111) surface and **h** S-terminated (111) surface. Views of the H absorbed on the **i** Ni-terminated (100) surface and **j** S-terminated (100) surface. **k** HER polarization curves of NiS_2_-octa and NiS_2_-cube. Reproduced with permission [82]. Copyright 2019, Elsevier Inc

Metal chalcogenide-containing metal sulfide and metal selenide are promising electrocatalysts for HER attributed to their earth abundance, high intrinsic catalytic activity, and abundant active sites [40, 41, 92, 110, 172, 173]. Taking the metal sulfide catalysts as an example, Liang et al. [82] presented a hydrothermal method to controllably prepared two NiS_2_ nanocrystals with exposed {111} and {100} planes, respectively (Fig. 9a-f). Based on DFT calculations, the surface energies of {111} and {100} planes were evaluated, which may offer a reasonable explanation for the difference in catalytic activity. The {111} planes of NiS_2_ exhibited higher surface energy compared to those of the {100} planes, indicating enhanced HER catalytic activity. Moreover, according to the proposed models (Fig. 9g-j), the adsorption energies of hydrogen atom on Ni-terminated and S-terminated {111} planes were calculated to be 10.67 and 2.42 eV, respectively, which were lower than those of the {100} planes (13.02 and 7.67 eV). The DFT results evidenced that {111} planes of NiS_2_ possessed stronger H adsorption capacity and better HER performance than {100} planes. {111} planes of NiS_2_ required an *η* of 138 mV to generate 10 mA cm^−2^ in 1.0 M KOH, while a much higher *η* of 302 mV was required for {100} planes of NiS_2_ (Fig. 9k). The {111} planes exhibited a Tafel slope of 139 mV dec^−1^, which was smaller than that of {100} planes (181 mV dec^−1^). Miao and co-workers [110] obtained mesoporous FeS_2_ via a facile synthetic protocol. The mesoporous structure and exposed {210} facets endowed FeS_2_ with a high specific surface area of 128 m^2^ g^−1^ and abundant accessible active sites, which could be beneficial for HER kinetics. Accordingly, the FeS_2_ catalyst achieved superior HER properties with a small Tafel slope of 78 mV dec^−1^, a low *η* of 96 mV at 10 mA cm^−2^, and long durability in 0.1 M KOH. DFT calculations confirmed that the abundant {210} facets of mesoporous FeS_2_ accounted for high HER performance and the low activation barrier.

**Fig. 10:**
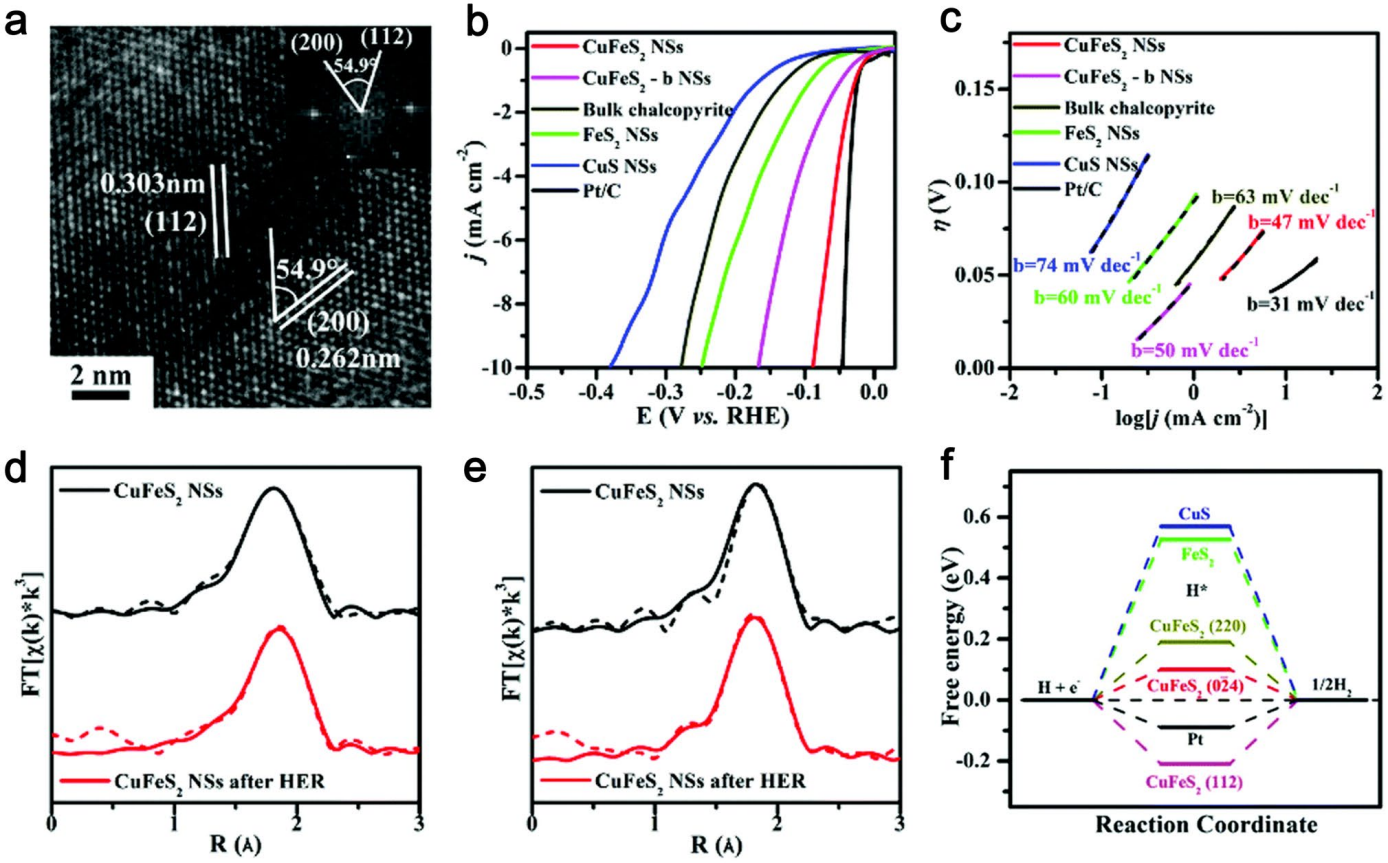
**a** HR-TEM image of CuFeS_2_ NSs. **b** HER polarization curves of CuFeS_2_ NSs, CuFeS_2_-b NSs, bulk chalcopyrite, CuS NSs, FeS_2_ NSs, and Pt/C. **d** Cu K-edge and **e** Fe K-edge before and after HER measurements, respectively. **f** The HER free energy diagram for three materials and Pt reference. Reproduced with permission [175]. Copyright 2017, Royal Society of Chemistry

Inspired by the periodic table where both S and Se are in group VIA, metal selenides were also expected as the potential electrocatalysts for HER [33, 100]. Zhong and coauthors developed a facile solid synthesis strategy for the preparation of RhSe_2_ with multiple facets of {200}, {210}, {211), {220}, and {311} [174]. DFT studies revealed that the ∆G_H_ of RhSe_2_ with multiple crystal facets was close to zero, suggesting abundant active sites in the RhSe_2_ catalyst. In acidic solution, low-coordinated Rh sites served as real active sites, which might allow the modified Kubas-mediated HER pathway to happen. Thus, the Kubas complexes were employed to overcome the high reaction barriers derived from the surface H diffusion (Tafel Reaction) as well as the proton–electron combination (Heyrovsky reaction) to produce hydrogen. In contrast, Se acted as real active sites and became more active in alkaline solution. Hydrogen evolution could proceed through the Volmer reaction termed rate-limiting step. Accordingly, the as-obtained RhSe_2_ with multiple active facets demonstrated the superior electrocatalytic property for HER with a small Tafel slope of 39 mV dec^−1^ and a low *η* of 49.9 mV at 10 mA cm^−2^ in 0.5 M H_2_SO_4_ as well as a *η* of 81.6 mV at 10 mA cm^−2^ and a Tafel slope of 86 mV dec^−1^ in 1.0 M KOH. In another work, Xi’s group successfully designed ultrathin metallic CuFeS_2_ nanosheets (CuFeS_2_ NSs) with the exposure of high-index {0–224} facets [175]. {112} facets and {200} facets are observed in Fig. 10a, wherein the angle between {112} facets and {200} facets was calculated to be 54.9° (closed to the theoretical value of 55°), indicating the existence of the exposed high-index {0–224} facets. As illustrated in Fig. 10b and c, the CuFeS_2_ NSs with the exposed high-index {0–224} facets achieved excellent HER performances with a small *η* of 88.7 mV (10 mA cm^−2^) and a low Tafel slope (47 mV dec^−1^) superior to those of CuFeS_2_-b with low-index {112} facets, bulk chalcopyrite, CuS NSs, and FeS_2_ NSs in 0.5 M H_2_SO_4_. To investigate the origin of enhanced HER catalytic activity, the HR-TEM test, XAS test, and DFT calculations of CuFeS_2_ NSs are performed, respectively. The HR-TEM results showed that {112} facets and {200} facets were still well preserved after the stability test, suggesting the existence of high-index {0–224} facets. As displayed in Fig. 10d, e, the coordination number of Cu and the R_Cu–S_ has hardly changed, while the coordination number of Fe decreased from 4.0 to 2.6. These results revealed that S^2−^ in the Fe–S site might be moved to the surface of the CuFeS_2_ NSs as active sites, leading to the decreased coordination number of Fe. Moreover, as presented in Fig. 10f, DFT results demonstrated the calculated ∆*G*_H_ on the high-index {0–224} facets is closest to zero compared to those of {112} facets and {200} facets, which further evidencing that high-index {0–224} facets feature abundant catalytic active sites for HER. Overall, the major cause of highly improved HER catalytic activity for the CuFeS_2_ NSs is related to the excess S^2−^ active sites on the exposed high-index {0–4} facets and the ultrathin nanosheet structure.

### Other Metal-Based Catalysts

**Fig. 11:**
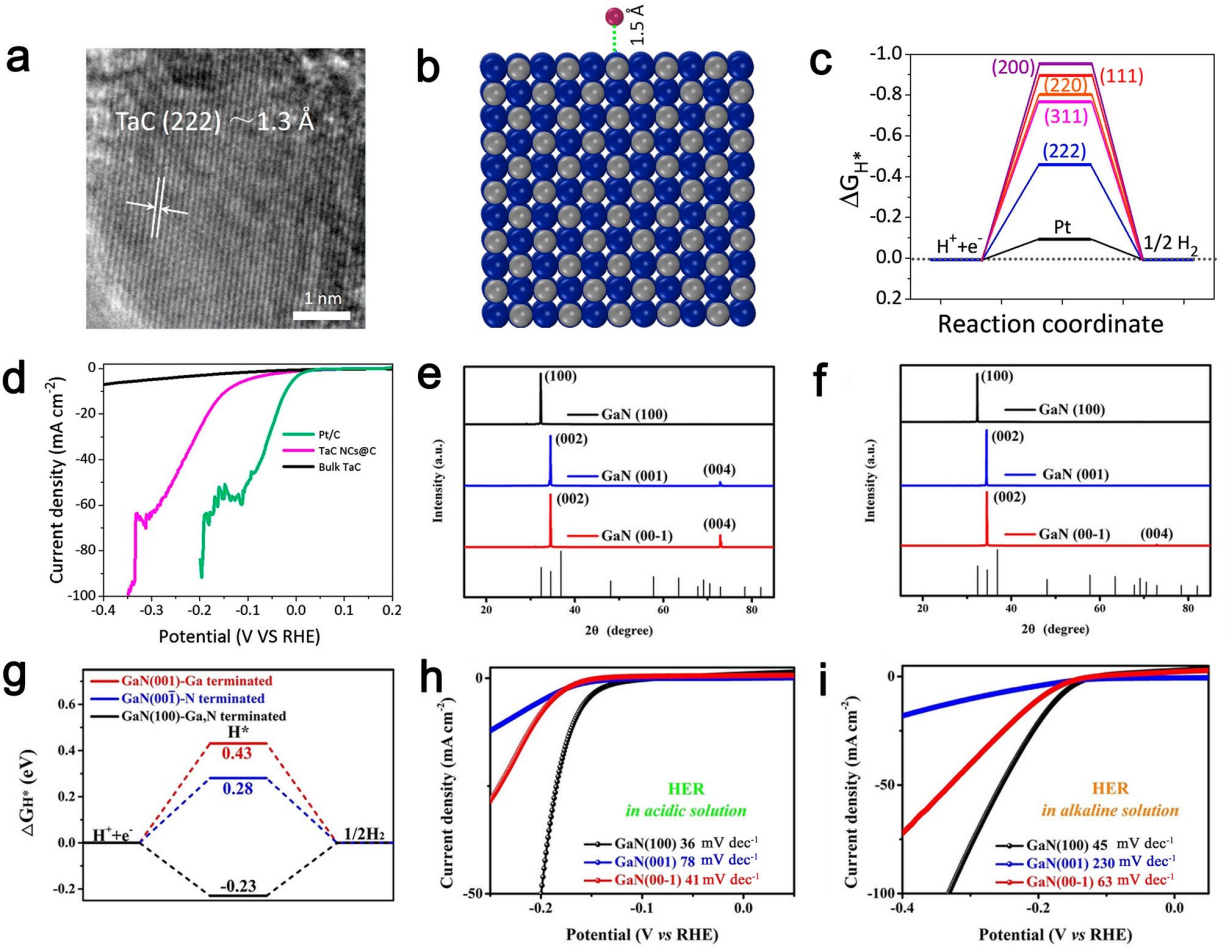
**a** HR-TEM image of TaC NCs@C. **b** Theoretical absorption model of H* atom on Miller index facets with an absorption distance of 1.5 Å. **c** DFT-calculated free energy diagram with different adsorption sites of H* at equilibrium potential for high-index (222) facet, relatively low-index (111), (200), (220), and (311) facets as well as Pt reference. **d** LSV curves of Pt/C, bulk TaC, and TaC NCs@C for HER. Reproduced with permission [94]. Copyright 2017, Elsevier Ltd. XRD patterns of GaN {001}, GaN {00-1}, and GaN {100} after HER durability tests in **e** acidic and **f** alkaline media. **g** DFT-simulated adsorption energies of H* on GaN (001), GaN (00-1), and GaN (100). HER polarization curves of GaN (001), GaN (00-1), and GaN (100) in **h** acidic and **i** alkaline solution. Reproduced with permission [73]. Copyright 2019, Wiley-VCH Verlag GmbH&Co

In addition to the above-mentioned catalysts, other transition metal-based compounds including metal carbides, metal nitrides, and metal oxides were also employed as high-performance HER catalysts. Metal carbides are highlighted by virtue of their high electronic conductivity, high catalytic activity and high stability toward HER [176, 177]. Mu’s group reported a “micro-cutting-fragmentation” technique to prepare carbon-cohered tantalum carbide nanocrystals (TaC NCs@C) with the exposure of the dominant high-index {222} facets by varying the reaction temperature (Fig. 11a) [94]. According to the calculated model, theoretical calculations unveiled the potential HER activity of {222} facets in TaC. Other low-index facets such as {311}, {220}, {200}, and {111} of TaC exhibited a ΔG_H_ value of -0.53, -0.56, -0.73, and -0.67 eV, respectively, which was all smaller than the value of the {222} plane (-0.23 eV), suggesting more active sites on {222} planes toward HER (Fig. 11b-c). The TaC NCs@C with exposed {222} planes produced at 950 °C showed a Tafel slope of 143 mV dec^−1^, reached 10 mA cm^−2^ at a η of 146 mV, and exhibited good stability in N_2_-saturated 0.5 M H_2_SO_4_ (Fig. 11d). Wang’s group constructed double-deck carbon-enveloped V_8_C_7_ networks with exposed highly active {110} planes supported on nickel foam (V_8_C_7_@GC NSs/NF) [178]. The V_8_C_7_@GC NSs/NF featured three exposed facets of typical {100}, {110}, and {111}. DFT results demonstrated that exposed {110} facets exhibited smaller ΔG_H_ value compared to those of {100} and {111} facets, indicating the enhanced intrinsic catalytic activity of {110} facets for HER. Therefore, the V_8_C_7_@GC NSs/NF with exposure of highly active {110} facets achieved distinguished catalytic activity toward HER with low *η* of 47, 77, and 38 mV at 10 mA cm^−2^ along with small Tafel slopes of 44, 64, and 34.5 mV dec^−1^ in 1.0 M KOH, 0.1 M PBS, and 0.5 M H_2_SO_4_, respectively.

Metal nitrides have attracted great attention on account of their unique physicochemical properties including high electronic conductivity, high chemical stability, and high electrocatalytic activity toward HER [179–181]. Xiao et al. [182] developed a facile CVD process to synthesis the face-centered cubic (FCC) 2D single-crystal TiN (FCC TiN) with exposed {111} facets. The FCC TiN exhibited a η of 210 mV at 10 mA cm^−2^ and a Tafel slope of 67 mV dec^−1^ toward HER in 0.5 M H_2_SO_4_. The major reason for the good HER activity was related to 2D single-crystal structure and exposed {111} facets. GaN crystals are considered as the ideal model for anisotropically exploring the reactive facets of electrocatalysts. Thus, Hu et al. [73] developed three GaN crystals with plane of {001}, {00-1}, and {100}, respectively. XRD patterns of the GaN crystal catalysts after HER durability tests in acidic and alkaline media are observed from Fig. 11e, f. Three GaN crystals with corresponding main crystal planes were consistent with the XRD results of the simulated GaN crystal catalysts, indicating distinguished chemical stability of GaN single-crystalline catalysts. The HER activity of corresponding three planes was theoretically and experimentally investigated. DFT calculations proved that the ΔG_H_ of exposed {100} plane was lower than those of {001} and {00–1} planes, demonstrating higher HER catalytic activity for {100} plane (Fig. 11g and Table 1). The electrochemical results also presented that the exposed reactive {100} planes required a η of 168 and 171 mV to produce a current density of 10 mA cm^−2^ in 0.5 M H_2_SO_4_ and 1.0 M KOH, respectively, which are superior to those of {001} (234 and 296 mV) and {00–1} planes (205 and 197 mV) at the same current density (Fig. 11h, i). The Tafel slope of {100} planes was approximately 36 mV dec^−1^ in acidic solution and 45 mV dec^−1^ in alkaline solution, which was smaller compared to the corresponding value of {001} planes (78 and 230 mV dec^−1^) and {00–1} planes (41 and 63 mV dec^−1^).

**Table 1:** Comparisons of HER/OER intrinsic properties according to some electrocatalysts depending on their facets

HER/OER/OWS electrocatalysts	Dominant facets	Hydrogen adsorption free energy (Δ*G*_H_, eV)	Theoretical overpotential (*η*, V)	References
GaN GaN GaN	{100}	− 0.23	/ / /	[73]
	{00-1}	0.28		
	{001}	0.43		
Pd@Ir TOH Pd@Ir cube Pd@Ir oct	{331}	/ / /	0.73	[191]
	{100}		0.99	
	{111}		1.47	
α-Fe_2_O_3_ α-Fe_2_O_3_ α-Fe_2_O_3_ α-Fe_2_O_3_	{012}-O	/ / / /	0.34	[70]
	{012}		0.57	
	{104}		0.95	
	{110}		1.55	
Co_3_O_4_ nanooctahedron	{111}	0.166	0.72	[221]
Co_3_O_4_ nanosheet	{112}	0.266	0.78	
Co_3_O_4_ nanobelt	{110}	0.291	1.04	
Co_3_O_4_ nanocube	{001}	0.419	1.12	
NiCo_2_O_4_ nanosheet NiCo_2_O_4_ octahedron NiCo_2_O_4_-truncated octahedron	{110}	0.15	0.65	[223]
	{111}	0.36	0.71	
	{100} {111}	0.62 0.36	0.91 0.71	

**Fig. 12:**
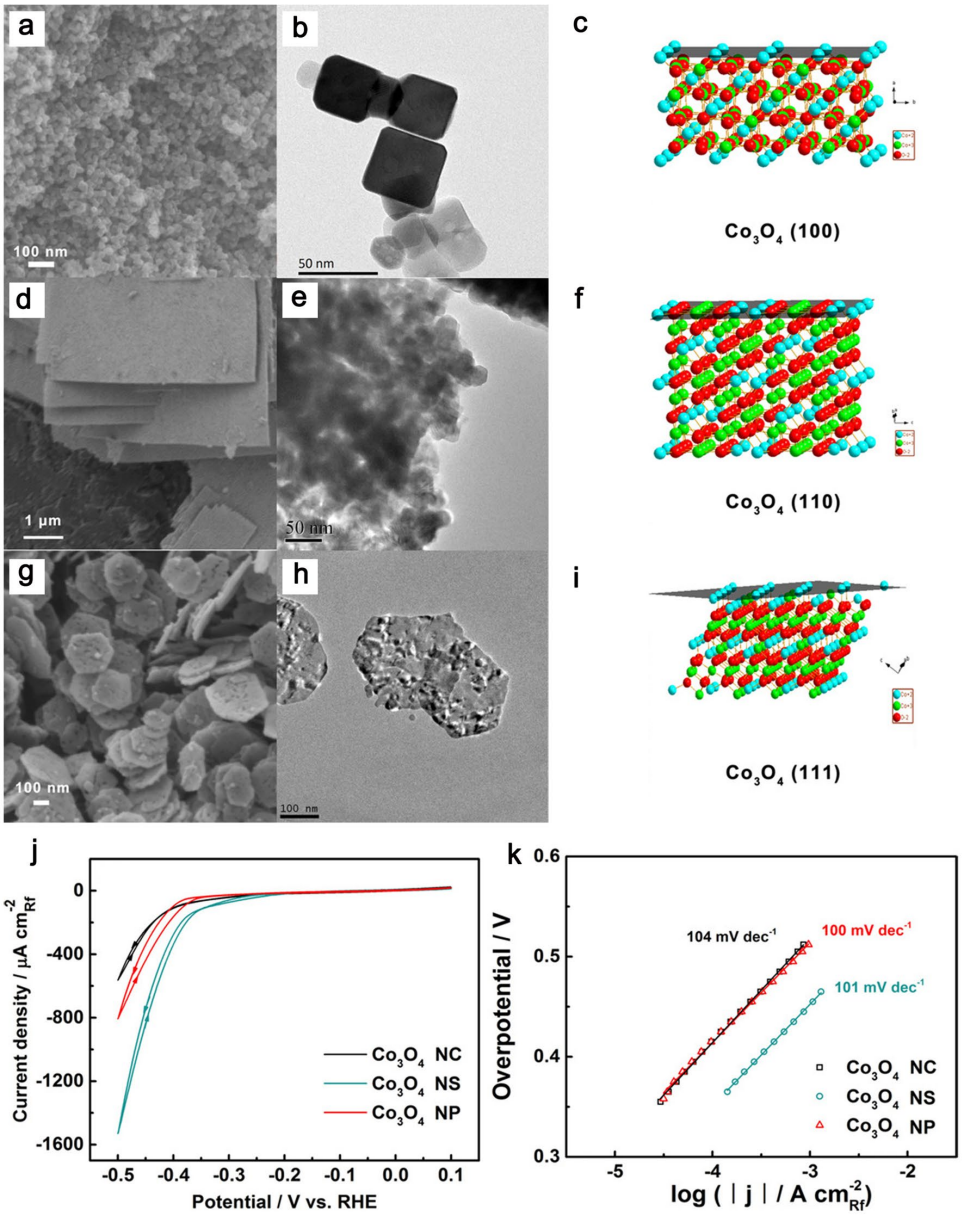
**a, d,** and **g** SEM images, **b, e,** and **h** TEM images, and **c, f,** and **i** structure models of Co_3_O_4_-NC, Co_3_O_4_-NS, and Co_3_O_4_-NP, respectively. **j** LSV curves of Co_3_O_4_-NC, Co_3_O_4_-NS, and Co_3_O_4_ for HER. **k** Tafel plots derived from panel **j**. Reproduced with permission [185]. Copyright 2018, Wiley-VCH Verlag GmbH & Co. KGaA, Weinheim

Benefitting from low cost, earth abundance as well structural and compositional diversity, metal oxides as the promising HER electrocatalysts have been attracted increasingly attention [183, 184]. Han and co-workers proposed the facile solvothermal and hydrothermal methods to fabricate three kinds of Co_3_O_4_ nanocube (Co_3_O_4_-NC) enclosed by {100} crystal planes, Co_3_O_4_ nanosheet (Co_3_O_4_-NS) bounded by {110} crystal planes, and Co_3_O_4_ nanoplate (Co_3_O_4_-NP) dominated by {111} crystal planes, respectively (Fig. 12a-i) [185]. The HER catalytic performances of three Co_3_O_4_ catalysts were measured in 0.1 M KOH electrolyte. The Co_3_O_4_-NS exhibited better HER catalytic activity in comparison with those of Co_3_O_4_-NC and Co_3_O_4_-NS, demonstrating that the HER catalytic properties of three exposed planes followed the order of {100} < {111} < {110} (Fig. 12j). As shown in Fig. 12k, the difference among the corresponding values of Tafel slopes was relatively small. The higher HER activity of Co_3_O_4_-NS might be accredited to higher electroconductivity and higher content of Co^3+^ ions on the surface of Co_3_O_4_-NS.

## Facet-Engineered Catalysts for OER

### Noble Metal Catalysts

**Fig. 13:**
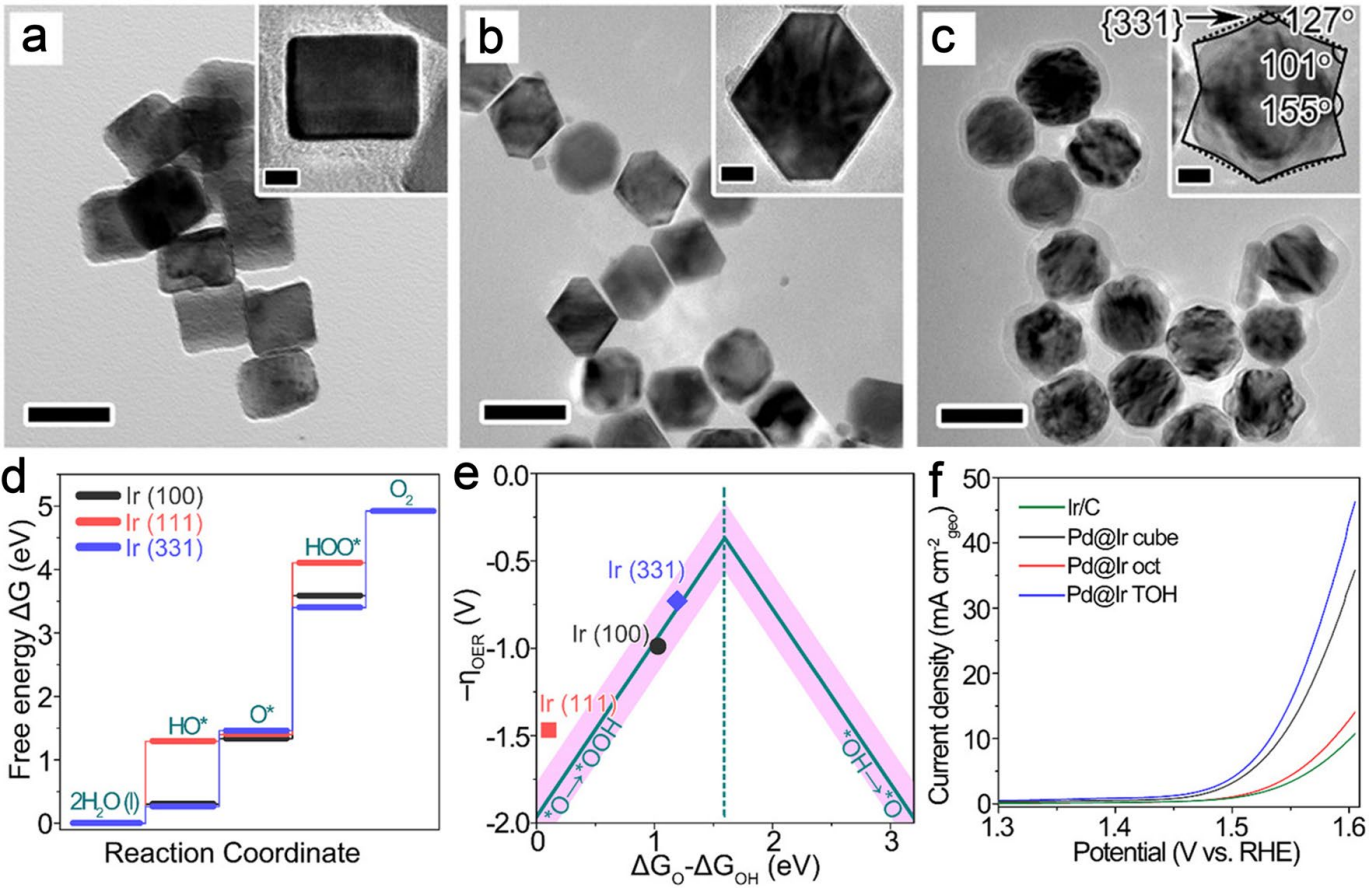
TEM images of **a** Pd@Ir cube, **b** Pd@Ir oct, and **c** Pd@Ir TOH (scale bar: 50 nm). **d** Free energy diagram of Ir (100) facets, Ir (111) facets, and Ir (331) facets, respectively. **e** Simulated η against the standard free energy of ΔG_O_-ΔG_OH_ on Ir (100), Ir (111), and Ir (331) facets. **f** OER polarization curves of Ir/C, Pd@Ir cube, Pd@Ir oct, and Pd@Ir TOH. Reproduced with permission [191]. Copyright 2021, American Chemical Society

Noble metal-based catalysts, especially Ru- and Ir-based nanomaterials, are recognized as the most promising electrocatalysts toward OER attributing to their high intrinsic catalytic activity [26, 186–190]. For example, Xue et al. [191] constructed Pd@Ir core–shell nanostructures with diverse shapes of cubes (cube), octahedron (oct), and trioctahedron (TOH) via a conformal deposition method. The Pd@Ir cube, Pd@Ir oct, and Pd@Ir TOH were dominated by exposed Ir {100}, Ir {111}, and high-indexed Ir {331} facets, respectively (Fig. 13a-c). According to the DFT calculation results, Pd@Ir TOH enclosed by high-indexed {331} facets indicated the highest uphill energy barriers named RDS for the overall OER, which is lower than those of the Pd@Ir cube with exposed {100} facets and the Pd@Ir oct with exposed {111} facets, demonstrating an outstanding OER behavior for the Pd@Ir TOH (Fig. 13d and Table 1). The standard free energy difference (∆G_O_—∆G_OH_) was performed to further evaluate OER performance. As a result, the value of ∆G_O_—∆G_OH_ of exposed {331} facets derived from the Pd@Ir TOH was calculated to be about 1.60 eV, which was higher than those of exposed {100} facets of the Pd@Ir cube and exposed {111} facets of the Pd@Ir oct, confirming the better OER catalytic activity of exposed {331} facets in the Pd@Ir TOH (Fig. 13e). The electrochemical results in HClO_4_ aqueous solution also evidenced that the Pd@Ir TOH with the exposure of high-indexed {331} facets exhibited good OER properties with a Tafel slope of 84.9 mV dec^−1^ and a η of 300 mV, which were lower than the corresponding value of Pd@Ir cubes (311.5 mV, 97.8 mV dec^−1^), Pd@Ir oct (356 mV, 110 mV dec^−1^), and commercial Ir/C (370 mV, 111.7 mV dec^−1^) (Fig. 13f). Zhang et al. [192] explored a series of Ag_2–*x*_O/FTO-*i* electrodes (*i* stands for the current density during the electrodeposition) via galvanostatic electrocrystallization. The optimized Ag_2–*x*_O/FTO-1 electrode enclosed by dominated {111} facets presented good catalytic activity toward OER with a Tafel slope of 47 mV dec^−1^ and a η of 417 mV at 10 mA cm^−2^ and good stability over 10 h in 0.1 M K_2_B_4_O_7_. The high OER activity of Ag_2–*x*_O/FTO-1 is on account of larger exposed high-activity {111} facets and more exposed Ag^+^/Ag^2+^ ions.

### Metal Oxide Catalysts

**Fig. 14:**
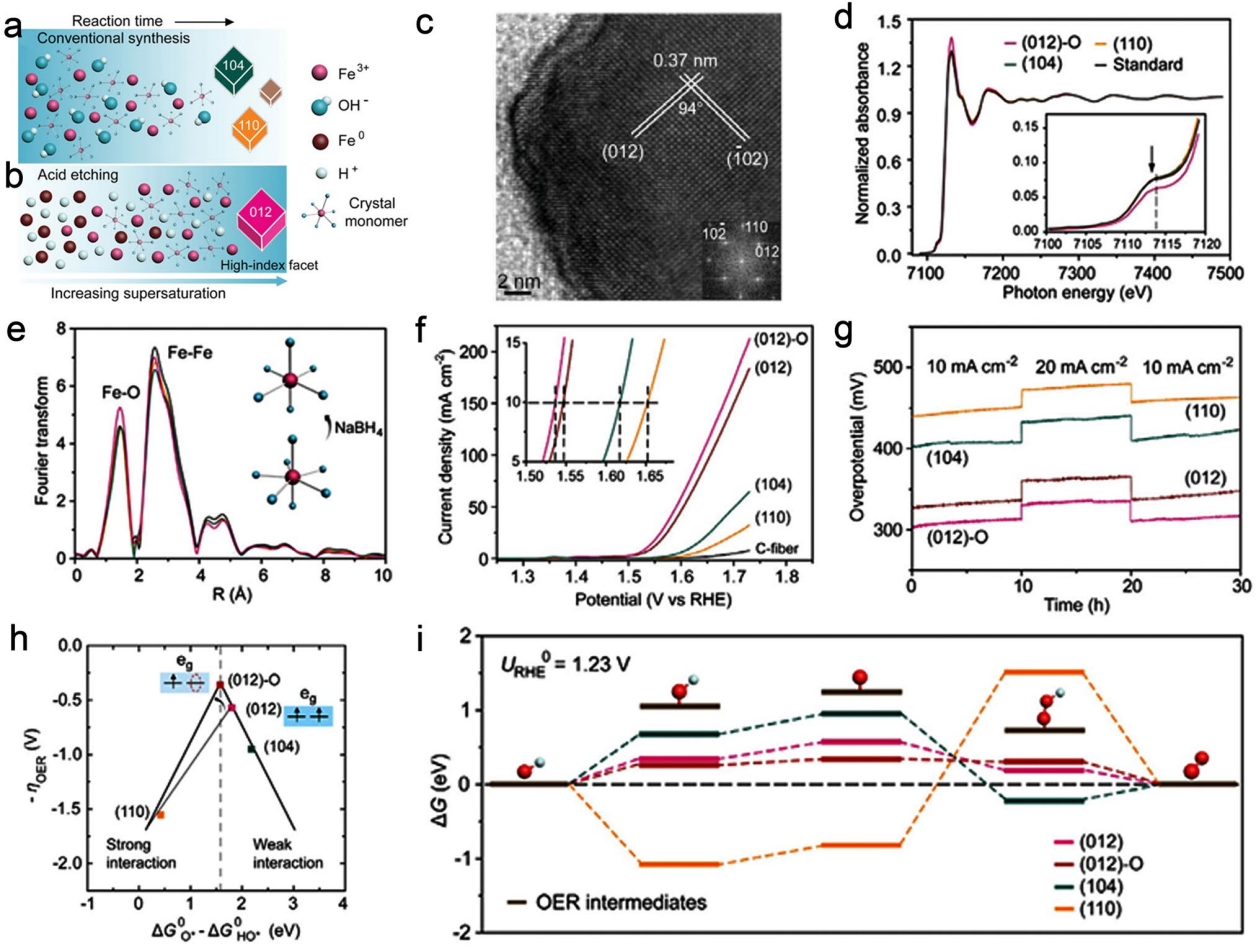
Schematic representation of α-Fe_2_O_3_ with different facets through **a** conventional solvothermal method and **b** acid-etching strategy. **c** HR-TEM image of the acid-etched α-Fe_2_O_3_. **d** XANES spectra of the three nanocrystals and standard hematite. **e** FT of EXAFS. Inset, schematic illustration of six- and seven-coordinated configurations. **f** OER polarization curves of C-fiber, (110) facets, (104) facets, (012) facets, and (104)-O facets. **g** Galvanostatic stability measurements. **h** Calculated *η* against the standard free energy of Δ*G*_O_–Δ*G*_OH_ on (110) facets, (104) facets, (012) facets, and (104)-O facets. **i** Free energy diagram for OER on (110) facets, (104) facets, (012) facets, and (104)-O facets at *U* = 1.23 V. Reproduced with permission [70]. Copyright 2018, Wiley-VCH Verlag GmbH & Co

A strategy known as selective etching of low-active crystal facets with retention of highly reactive crystal facets has shown to be effective in improving the OER intrinsic catalytic activity. Wu et al. [70] prepared three binary hematites (α-Fe_2_O_3_) enclosed by {104}, {110}, and {012}-O facets through the conventional solvothermal and hydrothermal methods as well as unconventional acid-etching strategy, respectively (Fig. 14a, b). The α-Fe_2_O_3_ was treated by HCl solution to produce {012}-O facets, which could be evidenced from the HR-TEM image in Fig. 14c. Subsequently, the acid-etched α-Fe_2_O_3_ was treated with NaBH_4_ solution to yield the {012} facets. In order to reveal the local environment, Fe K-edge (X-ray adsorption near edge structure) XANES spectra of α-Fe_2_O_3_ with {012}-O facets were performed using the XAS. As depicted in Fig. 14d, α-Fe_2_O_3_ with {012}-O facets demonstrated the lower intensity in terms of pre-edge peak at around 7114 eV. Besides, the corresponding Fourier transforms (FT) of extended X-ray absorption fine structure (EXAFS) were conducted to plot and fit intending to elucidate the local atomic and electronic structure (Fig. 14e). The XANES results illustrated that α-Fe_2_O_3_ with {012}-O facets possessed a higher coordination number (close to seven) of Fe–O, which reduces the e_g_ occupancy in the highly covalent Fe orbital and further strengthens the weak bonding of the OER intermediates during the OER process, thus boosting the intrinsic catalytic activity. The exposed high-indexed {012} facets endowed corresponding α-Fe_2_O_3_ with high OER activity in 1.0 M NaOH, requiring a η of 317 mV to drive 10 mA cm^−2^, which was lower compared to the value of {104} facets (388 mV) and {110} facets (422 mV) at the same current density (Fig. 14f). In addition, the Tafel slope of the {012} facets was 58.5 mV dec^−1^, which was lower than that of {104} facets (62.5 mV dec^−1^) and {110} facets (67.2 mV dec^−1^). The α-Fe_2_O_3_ with the {012}-O facets exhibited superior stability than those of {012}, {104}, and {110} facets, which are observed from Fig. 14g. DFT studies unveiled that bonding between {104} facets and the two intermediates (HO* and O*) was too weak, while the {110} facet was excessively strong, indicating unfavorable OER kinetics. The {012} facets at the apex of the volcano diagram exhibited a relatively more suitable binding energy, which was beneficial for a favorable intermediary adsorption/desorption and thus generated a lower reaction energy barrier (Fig. 14h-i). However, Fe sites of the seven-coordinated Fe centers endowed the acid-etched α-Fe_2_O_3_ with the extension of Fe–O bonds, which can strengthen the weak bonding of oxygenated intermediates and accelerate the OER kinetics during the OER process. Therefore, compared to other facets, the {012}-O facets delivered the best OER properties with a relatively low Tafel slope of 51.8 mV dec^−1^ and a smaller η of 305 mV at 10 mA cm^−2^.

The morphology of metal oxides can be manipulated for the preparation of dominant highly active facets to boost OER performances. Wang et al. [193] fabricated NiO nanobelts and nanoplates with dominant {110} and {111} planes, respectively. The NiO nanobelts with dominant {110} planes exhibited the good electrocatalytic OER performance with a η of 382 mV at 50 mA cm^−2^ superior to that of the NiO nanobelts with dominant {111} planes in 1.0 M KOH. DFT results demonstrated that the NiO nanobelts with dominant {110} planes exhibited the lower theoretical η (0.70 V) than {110} planes (1.70 V), illustrating the enhanced intrinsic electrocatalytic activity of {110} planes. The higher OER activity of {110} planes might be originated from the different crystallinities of NiO. Niu et al. [194] designed and synthesized four kinds of Fe_2_O_3_ (concave octahedral-Fe_2_O_3_ (CO–Fe_2_O_3_) with dominant {206} and {119} facets, rod-like Fe_2_O_3_ (RO-Fe_2_O_3_) with dominant {119} and {0012} facets, octahedron-like Fe_2_O_3_ (OC-Fe_2_O_3_) with dominant {119} and {0012} facets, and spindle-like Fe_2_O_3_ (SP-Fe_2_O_3_) with dominant {203} facets) by pyrolyzing a series of FeO_X_ node-based MOFs. The 3D porous structure endowed CO-Fe_2_O_3_ with fast charge/mass transport, which could be beneficial for OER kinetics. Importantly, the CO-Fe_2_O_3_ featured the highly active facets of {206} and {119}, which could effectively boost the intrinsic catalytic activity. As a result, the CO-Fe_2_O_3_ exhibited better catalytic activity with a η of 439 mV and a Tafel slope of 99 mV dec^−1^ which are better than those of RO-Fe_2_O_3_ (478 and 104 mV dec^−1^), OC-Fe_2_O_3_ (533 and 115 mV dec^−1^), and SP-Fe_2_O_3_ (575 and 120 mV dec^−1^) in N_2_-saturated 1.0 M KOH solution.

Similarly, Liu et al.[90] reported a hydrothermal strategy to fabricate CoMoO_4_ nanorods (NR) predominantly enclosed by {100} facets and CoMoO_4_ nanosheets (NS) mainly bounded by {010} facets. The CoMoO_4_ NR required a η of 550 mV to yield 8.93 ${\mathrm{mA}}_{\mathrm{cat}}^{-2}$, lower than the value of the CoMoO_4_ NS (1.56 ${\mathrm{mA}}_{\mathrm{cat}}^{-2}$) at the same η. The major cause of the higher intrinsic OER activity of CoMoO_4_ NR was related to exposed {100} facets and thus abundant Co active sites at {100} facets. Besides, DFT studies demonstrated that the {100} facets presented a higher surface energy of 0.54 J m^−2^ than 0.41 J m^−2^ for {010} facets, indicating {100} facets were more reactive toward OER. Wang and co-workers synthesized ferric vanadate (FeVO_4_) nanosheets and nanobelts dominated by {001} and {010} facets, respectively [195]. The open surface structure and countless catalytic active sites of Fe and V on the {010} facets can enhance the electrocatalytic activity of FeVO_4_ nanobelts toward OER with superior durability over 36 h continuous electrolysis, a small Tafel slope of 37.4 mV dec^−1^ and a low η of 240 mV to obtain 10 mA cm^−2^ in 1.0 M KOH electrolyte.

### Metal Selenide Catalysts

**Fig. 15:**
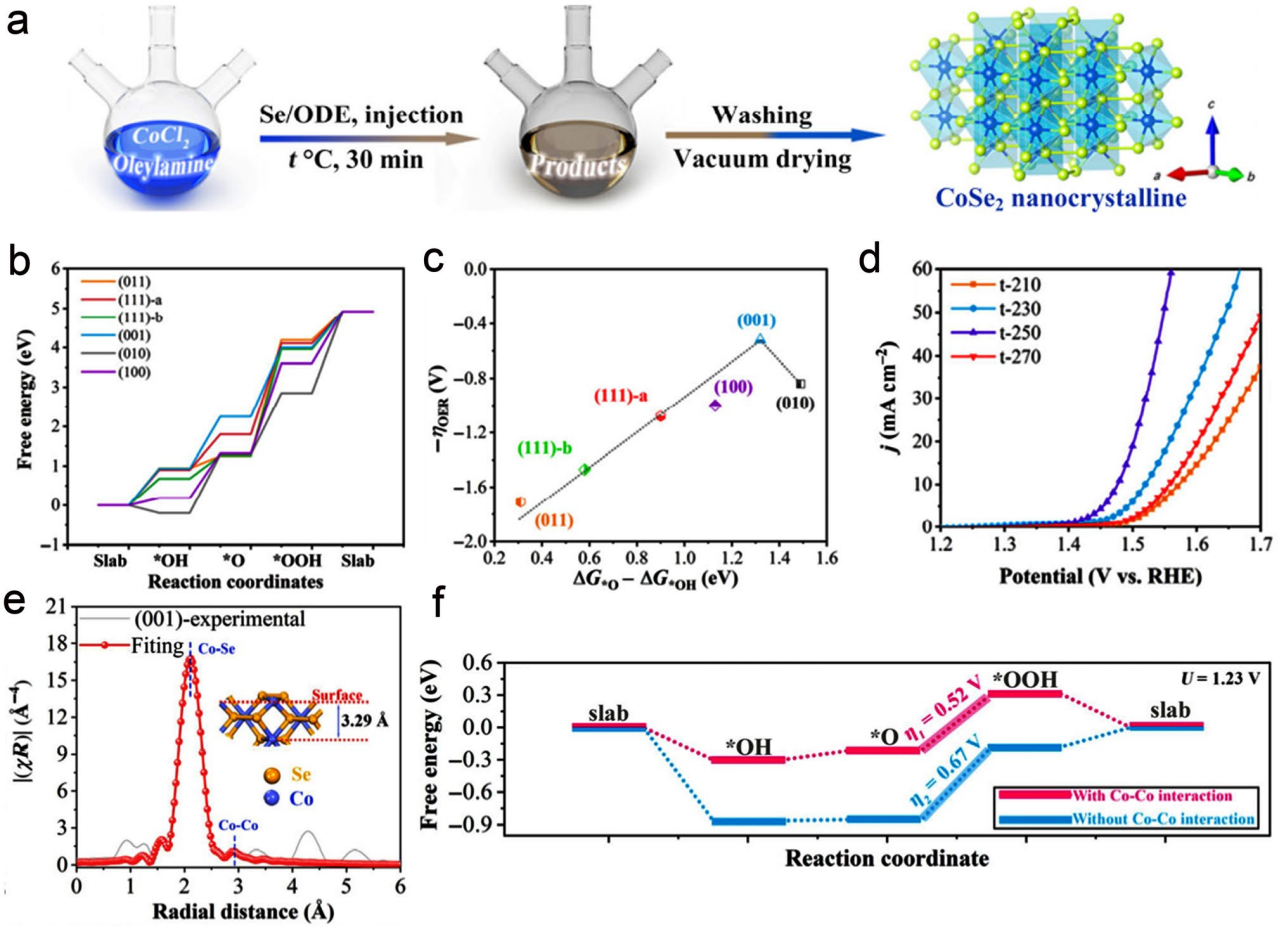
**a** Synthesis of *o*-CoSe_2_ nanocrystalline. **b** Reaction energy diagrams of the OER on (011) facets, (111)-a facets, (111)-b facets, (001) facets, (010) facets, and (100) facets, respectively. **c** Volcano plots for the OER overpotential on different crystal planes from **b**. **d** LSV curves of t-210, t-230, t-250, and t-270 for OER. Reproduced with permission [198]. Copyright 2021, Tsinghua University Press and Springer-Verlag GmbH Germany

The metal selenides have been widely developed as high-efficiency OER electrocatalysts thanks to their special layered architecture, relatively narrow bandgap and high intrinsic catalytic activity [33, 196, 197]. Dang et al. [198] proposed a high-temperature liquid-phase route to construct CoSe_2_ nanosheets with dominant {001} facets (Fig. 15a). The exposure ratio of {001} facets can be tailored through varying the temperature. Apart from {001} facets, CoSe_2_ nanosheets also featured other crystal facets including {111}-a, {111}-b, {011}, {010}, and {100} facets (Fig. 15b). The OER activity of these crystal facets was investigated by the DFT calculation, and volcano plots of η for corresponding crystal facets were further performed to screen desired facets with relatively high intrinsic catalytic activity (Fig. 15c). As expected, CoSe_2_ nanosheets with the exposure of {001} facets showed the better OER performance than CoSe_2_ with other facets, requiring a η of 240 mV to drive a current density of 10 mA cm^−2^ (Fig. 15d). The corresponding Tafel slope is 75.8 mV dec^−1^. Interestingly, the XAS is used to study the coordination environment of Co in the CoSe_2_ {001}. A small peak of about 2.9 Å, corresponding to the fitting length of 3.23 Å, is observed in Fig. 15e, which is very close to Co–Co path (3.29 Å) around the surface of the slab of *o*-CoSe_2_ {001}. The XAS results demonstrated that shorter Co–Co scattering path was a result of the shrinkage of the surface owing to the absence of Se atoms. The electronic structures of active sites can be tailored by dynamic short-range Co–Co interaction, which can improve OER catalytic performances. The DFT calculations further proved that shortened distance between Co atoms could enhance the electropositivity of active sites and tailor electronic structure, thus reducing the energy barrier of OER (Fig. 15f). In another study, Cai et al. [199] synthesized the dendrite-like nickel selenide (NiSe_2_) with highly active {200} facets at different temperatures. The obtained NiSe_2_ at 413 K disclosed good stability and OER activity with a Tafel slope of 71 mV dec^−1^ and a η of 299 mV at 10 mA cm^−2^ in 1.0 M KOH. The higher catalytic activity of NiSe_2_ toward OER can be mainly ascribed to the dendrite-like nanostructures along with high exposure ratio of {200} facets, resulting in high utilization ratio of active Ni sites.

### Metal Hydroxide/Oxyhydroxide Catalysts

**Fig. 16:**
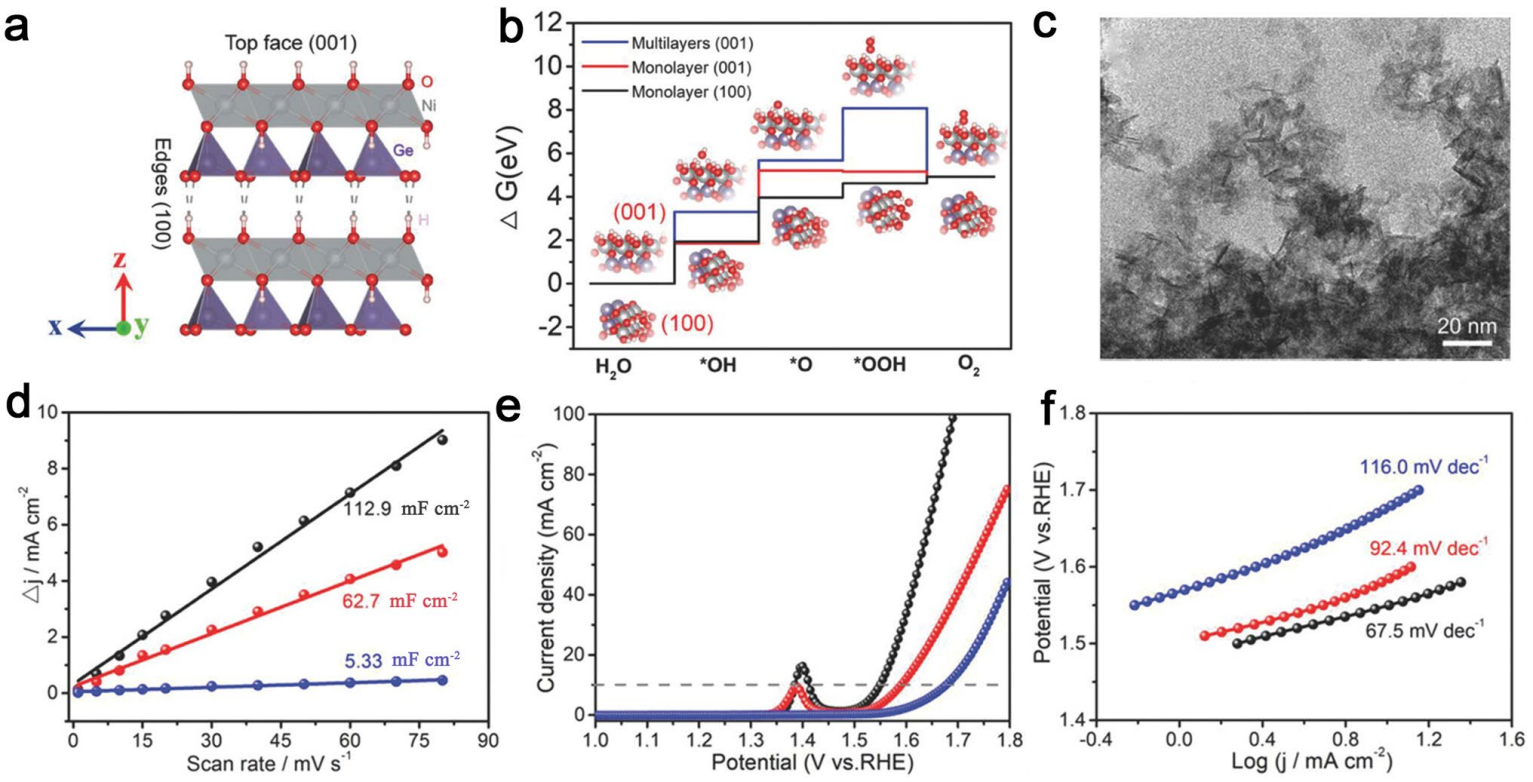
**a** Crystal structure of a Ni_3_Ge_2_O_5_(OH)_4_ nanosheet with (001) on top face and (100) on edges. **b** Standard free energy diagrams of OER intermediates on (001) facet in multilayered slab, (001) facet in monolayer slab, and (100) facet in monolayer slab. **c** TEM image of monolayered nanosheets. **d** Current density differences plotted against scan rates. **e** Polarization curves, and **f** Tafel plots of monolayered nanosheets (black plots), five-layered nanosheets (red plots), and ten-layered nanosheets (blue plots). Reproduced with permission [206]. Copyright 2018, Wiley-VCH Verlag GmbH & Co

Layered double metal hydroxides (LDHs) usually contain metallic divalent cations (M^II^), metallic trivalent cations (M^III^) as well as interlayered anions (A^n−^), which are written as M^II^_1-*x*_M^III^_*x*_(OH)_2_(A^*n*−^)_*x*/*n*_·*y*H_2_O [200–203]. The layered structures of LDHs are utilized to expose more active facets, thereby enhancing the intrinsic catalytic activity [204, 205]. For example, Zhang et al. [206] synthesized a series of layered serpentine Ni_3_Ge_2_O_5_(OH)_4_ nanosheets. According to the proposed model (Fig. 16a), the theoretical calculations confirmed that {100} facets in a monolayered slab possessed lower Gibbs free energy to oxygenated intermediates than those of {001} facets in a monolayered slab and {001} facets a multilayered slab, indicating the highly active {100} facets for OER (Fig. 16b). The sheet-like morphology of Ni_3_Ge_2_O_5_(OH)_4_ was observed from the TEM image in Fig. 16c. The Ni_3_Ge_2_O_5_(OH)_4_ with monolayered nanosheet possesses a higher electrochemical active surface area (ECSA), thus ensuring the higher electrocatalytic activity. Thus, synthesis thin layer and small size of Ni_3_Ge_2_O_5_(OH)_4_ can increase exposure of {100} facets and further add active sites, which is beneficial to boost OER catalytic performance. Monolayered Ni_3_Ge_2_O_5_(OH)_4_ nanosheet with highly exposed {100} facets demonstrated a low η of 320 mV to reach 10 mA cm^−2^ and a Tafel slope of 67.5 mV dec^−1^ (Fig. 16e, f). Zhao et al. [207] designed hierarchal NiFe LDH nanosheet-arrays-on-microplates (NiFe NSAs-MPs) with abundant exposed edge planes on nickel foam as high-efficiency OER electrocatalysts via a plane engineering method. Compared to NiFe LDHs microsheet arrays (NiFe MSAs) bounded by traditional {003} planes, the NiFe NSAs-MPs featured exposed edge planes of {012}, {015}, and {110} planes. The NiFe NSAs-MPs demonstrated an outstanding OER performance with a η of approximately 250 mV at 100 mA cm^−2^ as well as a small Tafel slope of 34.5 mV dec^−1^ in 1.0 M KOH, which is lower than that of the NiFe MSAs (300 mV and 63.0 mV dec^−1^). The theoretical calculation disclosed that the Fe sites were recognized as the active reaction site instead of the Ni sites. The results of volcano plot showed that the Fe sites as active reaction sites on the {012} planes illustrated a lower η than those of other planes, indicating that {012} plane was the most active plane, which could account for the high OER catalytic performance of NiFe NSAs-MPs.

### Metal–Organic Framework Catalysts

**Fig. 17:**
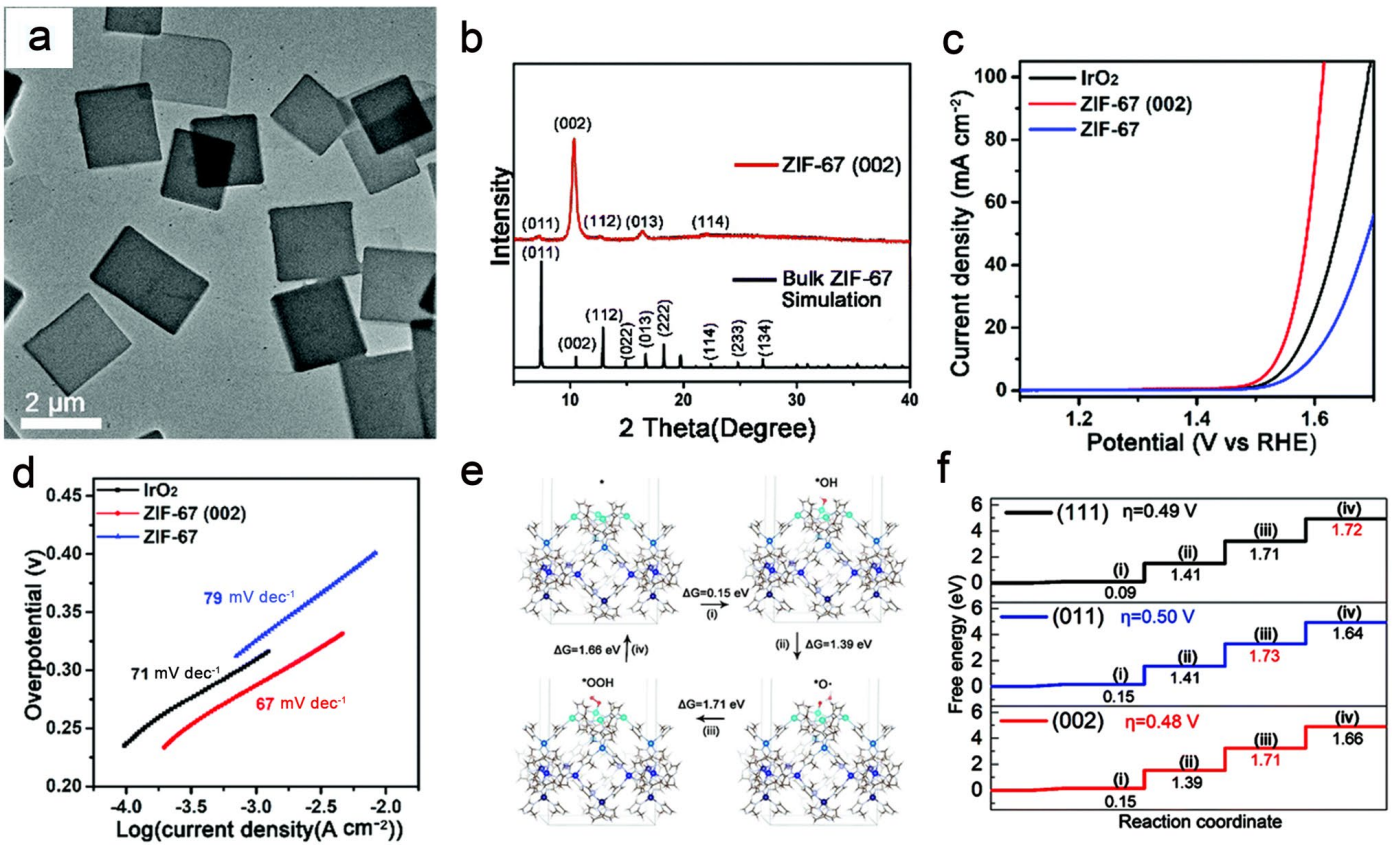
**a** TEM image and **b** XRD pattern of ZIF-67 {002} facets. **c** LSV curves and **d** Tafel plots of IrO_2_, ZIF-67 {002}, and ZIF-67. **e** Four energy steps of the OER process on ZIF-67 {002} surface. **f** Reaction energy diagrams of the OER for the {002}, {011}, and {111} facets, respectively. Reproduced with permission [80]. Copyright 2020, Royal Society of Chemistry

MOFs are assembled by metal nodes and organic linkers through coordination bond [43–45, 208–214]. MOFs generally feature well-defined and anisotropic crystal facets, which may lead to different electrocatalytic activity [46]. Thus, enlarging the proportion of active facets on the MOF surface can boost the intrinsic catalytic activity. Wan et al. [80] utilized sodium dodecyl sulfate (SDS) to reduce adsorption energies of specific facets during the ZIF-67 growth process and produced 2D ZIF-67 nanosheets with high exposure of the {002} facets (Fig. 17a, b). The as-obtained 2D ZIF-67 generated 10 mA cm^−2^ at a η of 305 mV along with a Tafel slope of 67 mV dec^−1^ (Fig. 17c, d). In order to investigate why 2D ZIF-67 exhibited high catalytic performances toward OER, as shown in Fig. 17e, f, the DFT calculations were performed and the results revealed that {002} facets exhibited a lower η of 0.48 V than those of {011} and {111} facets. Zhao et al. developed a new and facile coordination modulation method to achieve accurate construction of 2D NiFe-MOFs with dominant exposed {001} facets [84]. Specifically, acetate ions were selected as the coordination modulators to fabricate ultrathin NiFe-MOFs nanosheets with mainly exposure of {001} facets, while the bulk NiFe-MOF was obtained without the coordination modulator. The highly exposed {001} facet of the 2D NiFe-MOFs offered abundant active sites, enhanced mass/charge transport and accelerated surface kinetics. The 2D NiFe-MOFs with mainly exposed {001} facets disclosed high OER properties with a Tafel slope of 73.4 mV dec^−1^ and an attractive η of 240 mV at 10 mA cm^−2^ and superior stability over 16 h in 1.0 M KOH.

## Facet-Engineered Bifunctional Catalysts for the Water Splitting

The simultaneous occurrence of the HER and OER under the same electrolyte is the prerequisite for practical electrolysis of water [13, 22]. Unfortunately, HER catalysts usually exhibit remarkable electrocatalytic activity in strong acidic solution, while OER catalysts typically demonstrate distinguished catalytic performances in strong alkaline solution. The mismatching of working conditions is an inevitable concern in searching for water splitting catalysts [215]. Importantly, the true catalytically active sites of the HER and OER are inconsistent. For example, reconstruction phenomena usually occur on the surface of metal oxides/phosphides/sulfides during anodic OER polarization, resulting in the formation of the real active sites [117, 216–219]. Due to the differences between HER and OER catalytic mechanisms, design high-efficiency bifunctional water splitting catalysts is a challenging project [220]. Crystal facet engineering has been regarded as the effective method to obtain dominant exposed highly active crystal facets. These crystal facets featured with abundant unsaturated coordination sites would present superior water splitting activity. Therefore, in the following, in-depth discussions about facet-engineered water splitting catalysts of metal oxides and sulfides are given as the typical examples.

### Metal Oxide Catalysts

**Fig. 18:**
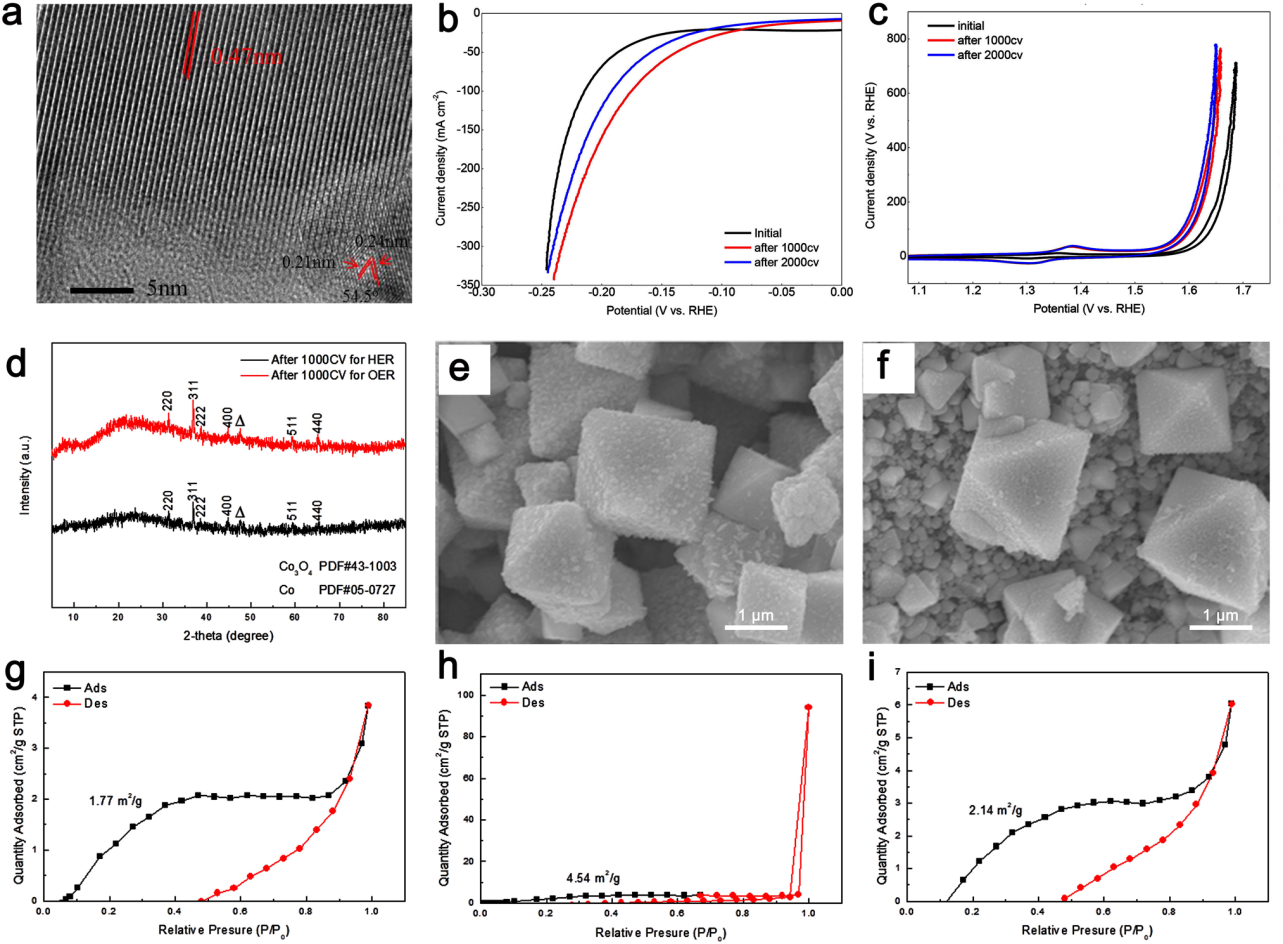
**a** HR-TEM image of the octahedral Co_3_O_4_ particles. **b** HER and OER, **c** polarization curves before and after continuous potential sweeps at 50 mV s^−1^. **d** XRD patterns of the octahedral Co_3_O_4_ particles after stability test of 1000 cycles. **e, f** SEM images of the octahedral Co_3_O_4_ particles after stability test of 1000 cycles toward **e** HER and **f** OER. BET adsorption and desorption isotherm for **g** octahedral Co_3_O_4_ particles, **h** octahedral Co_3_O_4_ particles after HER stability test, and **i** octahedral Co_3_O_4_ particles after OER stability test. Reproduced with permission [222]. Copyright 2018, Elsevier Ltd

Pure metal oxides, especially the bulk materials, have not demonstrated satisfactory HER performances attributed to their poor electrical conductivity, which hinders the development of metal oxides for electrocatalytic water splitting [183, 184]. Thus, various strategies of doping, oxygen vacancy engineering, surface reconstruction, and facet engineering have been proposed to boost the HER activity [183]. Intriguingly, metal oxides constructed by facet engineering exhibit comparable catalytic performances toward both HER and OER. Liu et al. [221] successfully prepared four morphological Co_3_O_4_ catalysts including nanocube bounded by {001} facets, nanobelt enclosed by {110} facets, nanooctahedron dominated by {111} facets, and nanosheet with exposed {112} facets. The electrochemical properties of these facets for OER, HER, and OWS were systematically explored in 1.0 M KOH. The {111} facets achieved better OER performances with the smallest η of 285 mV to afford a current density of 10 mA cm^−2^ compared to those of {001} facets (362 mV), {110} facets (341 mV), and {112} facets (312 mV). The {111} facets exhibited a Tafel slope of 49 mV dec^−1^, which was much lower than those of {001} facets (119 mV dec^−1^), {110} facets (101 mV dec^−1^) as well as {112} facets (73 mV dec^−1^). Moreover, the {111} facets delivered better catalytic activity toward HER with a smaller η of 195 mV superior to those of {001} facets (284 mV), {110} facets (260 mV), and {112} facets (232 mV). The corresponding Tafel slopes of {111}, {001}, {110}, and {112} facets were 47, 97, 78, and 59 mV dec^−1^. The Co_3_O_4_ nanooctahedron system ({111}||{111}) needed a potential of 1.60 V to reach 10 mA cm^−2^ toward OWS, which was much lower than those of {001}||{001}(1.74 V), {110}||{110}(1.69 V), and {112}||{112}(1.65 V). DFT calculations were used to reveal the correlation between electrocatalytic activity and crystal facets (Table 1). The results demonstrated that the {111} facets possessed the highest surface energy, the biggest dangling bond density as well as the smallest absolute value of Δ*G*_H_ than those of other facets, which were responsible for the superior catalytic activity of {111} facets for both the OER and HER. Wu et al. proposed a hydrothermal synthesis of octahedral Co_3_O_4_ particles grown on cobalt foam [222]. An interplanar distance of 0.47 nm in Fig. 18a corresponds to the high-index {111} facets of the Co_3_O_4_, which is proven to feature satisfactory catalytic performances toward HER and OER. The octahedral Co_3_O_4_ particles with exposed high-index {111} facets displayed good HER, OER, and OWS performances with the η of 77.9, 301.2, and 370 mV to drive the current density of 10 mA cm^−2^ in 1.0 M KOH, respectively. The high catalytic activity of the octahedral Co_3_O_4_ particles mainly originates from the exposure of a large number of high-index {111} facets as well as the existence of rich hydroxyl groups on cobalt oxide surface. Interestingly, as shown in Fig. 18b, c, after CV stability test, the catalytic performances of the octahedral Co_3_O_4_ particles for both HER and OER are improved. To investigate the reason of highly improved catalytic activity, The XRD, SEM, and BET tests to characterize the octahedral Co_3_O_4_ particles are performed. The XRD result showed the main phase peaks of the octahedral Co_3_O_4_ particles after HER/OER stability tests are well preserved (Fig. 18d). The surface of the octahedral Co_3_O_4_ particles became significantly roughened after HER/OER stability tests (Fig. 18e, f). The results of BET tests illustrated that the increased specific surface area of the octahedral Co_3_O_4_ particles after HER/OER stability test is observed from Fig. 18g-i, which can expose more electrochemically active sites and thus enhance electrocatalytic activity toward HER and OER.

**Fig. 19:**
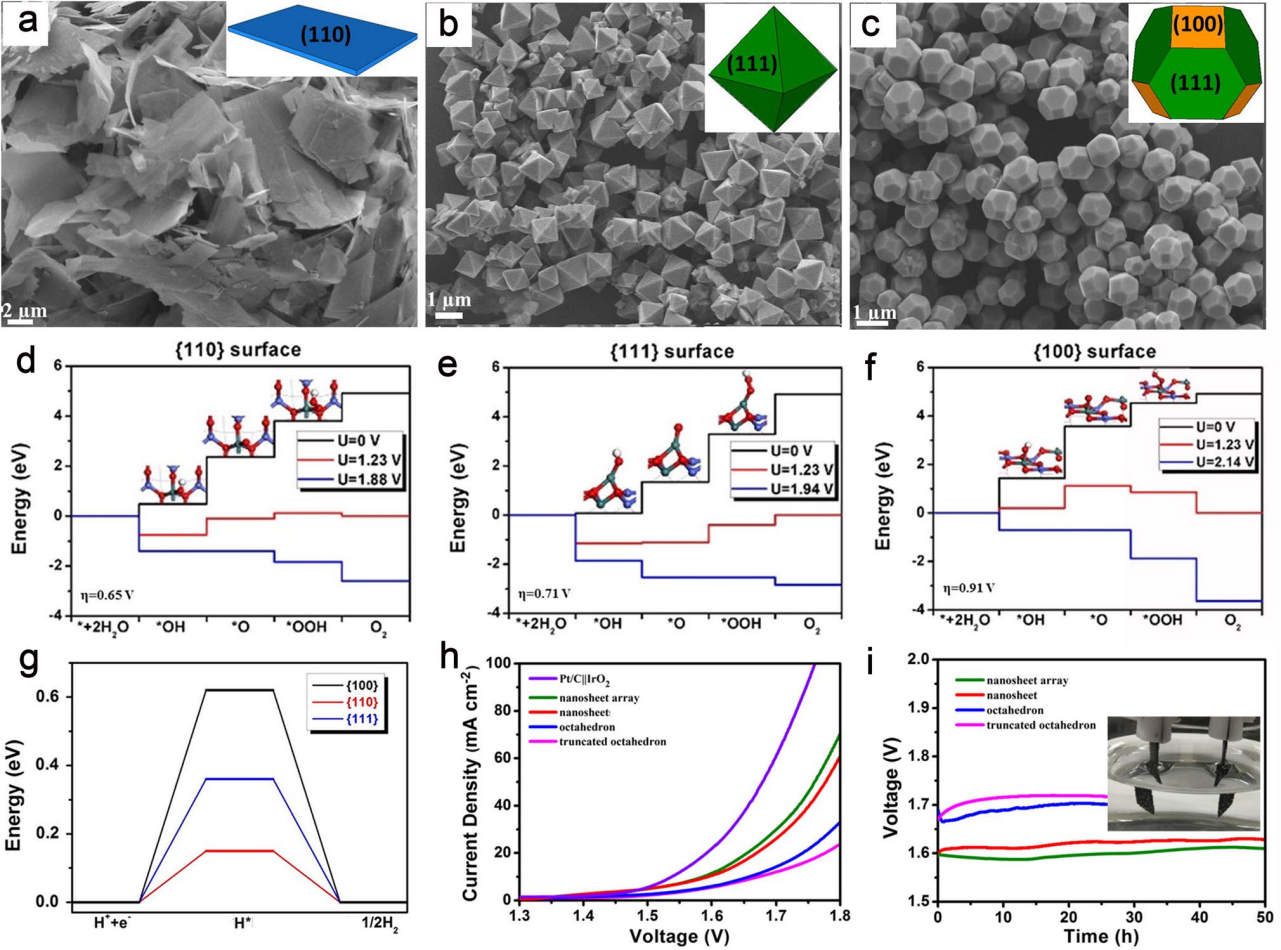
SEM images of **a** NiCo_2_O_4_ nanosheets with the exposed {110} facet, **b** NiCo_2_O_4_ octahedrons enclosed by {111} facet, and NiCo_2_O_4_-truncated octahedrons bounded by {111} and {100} facet, respectively. Reaction energy diagrams of OER for **d** the {110}, **e** the {111}, and **f** the {100} facet. **g** Free energy diagram of {100}, {110}, and {111} for HER, respectively. **h** LSV curves of the OWS for a series of NiCo_2_O_4_ catalysts and Pt/C||IrO_2_. **i** Long-term stability measurement for a series of NiCo_2_O_4_ catalysts at 10 mA cm^−2^. Reproduced with permission [223]. Copyright 2017, Elsevier Inc

In another typical example, Fang et al. [223] proposed a simple and general method of controlling the pH of the solution for the preparation of NiCo_2_O_4_ nanosheet enclosed by {110} crystal planes, NiCo_2_O_4_ octahedron bounded by {111} crystal planes as well as NiCo_2_O_4_-truncated octahedron dominated by {111} and {100} crystal planes, respectively (Fig. 19a-c). Theoretical calculations were performed to evaluate catalytic activity of these crystal planes. As illustrated in Fig. 19d-g and Table 1, compared to {111} planes and {100} planes, the {110} planes exhibited smaller ΔG_H_ for HER and lower energy barrier for OER as well as higher surface energy for adsorption of ionized oxygen species. The results evidenced that the catalytic performances of three exposed facets for HER and OER followed the order of {110} > {111} > {100}. The NiCo_2_O_4_ nanosheet required a η of 157 mV to drive 5 mA cm^−2^, which is lower than the value of NiCo_2_O_4_ octahedron (197 mV) and NiCo_2_O_4_-truncated octahedron (223 mV). The corresponding Tafel slopes were 71.2, 93.8, and 110.3 mV dec^−1^. The NiCo_2_O_4_ nanosheet illustrated better OER activity with a smaller potential of 1.56 V and a lower Tafel slope of 59.2 mV dec^−1^ compared to the values of NiCo_2_O_4_ octahedron (1.59 V and 78.1 mV dec^−1^) and NiCo_2_O_4_-truncated octahedron (1.63 V and 86.9 mV dec^−1^). The NiCo_2_O_4_ nanosheet displayed excellent electrocatalytic stability toward OWS and reached 10 and 20 mA cm^−2^ at 1.59 and 1.65 V, respectively, which were smaller than the value of NiCo_2_O_4_ octahedron (1.60 and 1.67 V) and NiCo_2_O_4_-truncated octahedron (1.66 and 1.74 V) at the same current density (Fig. 19h, i).

### Metal Sulfide Catalysts

**Fig. 20:**
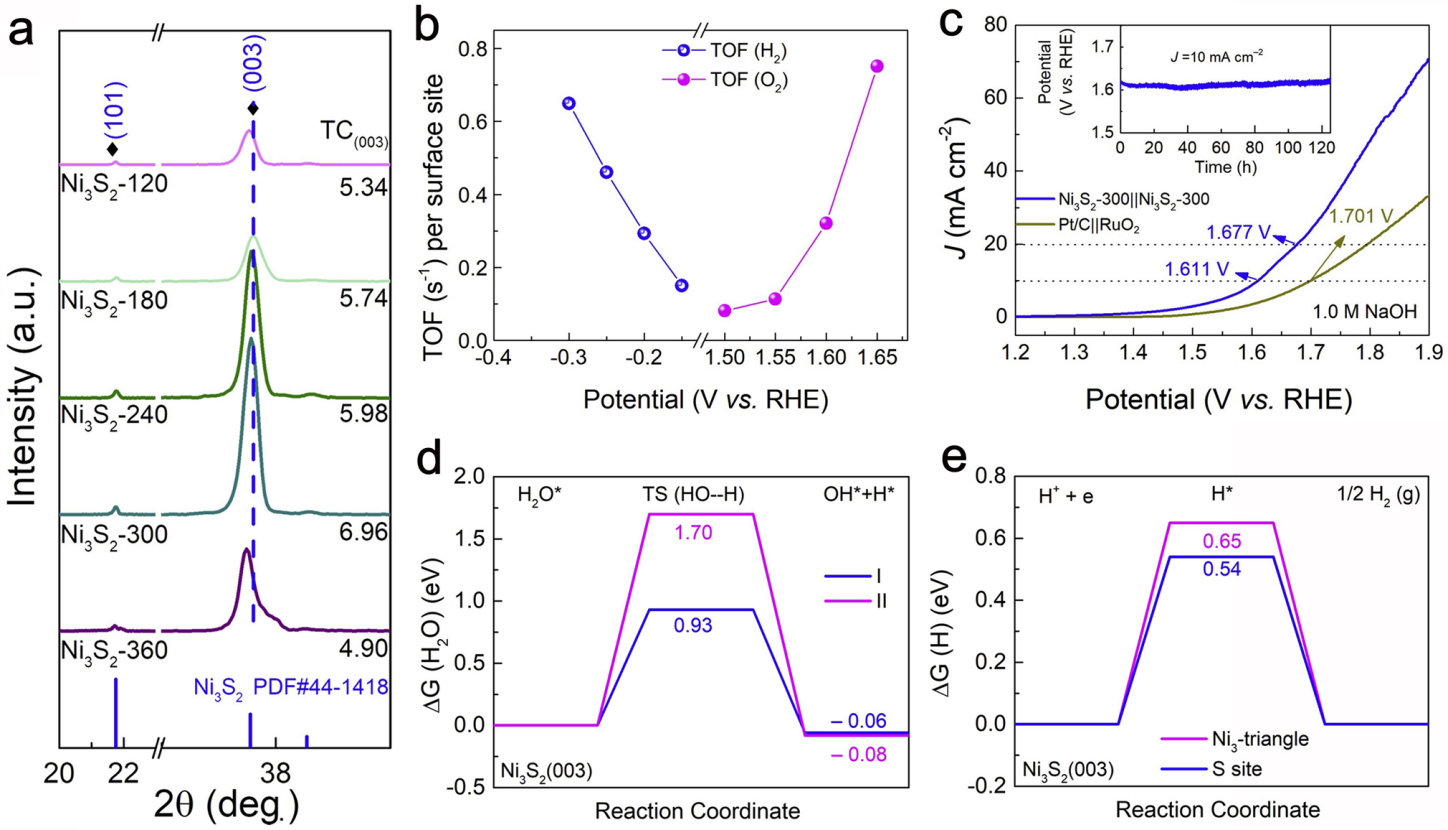
**a** XRD patterns of Ni_3_S_2_ with the different sulfurization time. **b** TOFs of Ni_3_S_2_-300 plotted against potential for HER and OER. **c** LSV curves of the OWS for the Ni_3_S_2_-300||Ni_3_S_2_-300 and Pt/C||Ru_2_O catalysts. Calculated adsorption free energy diagrams for the **d** Volmer step and **e** Tafel step on the Ni_3_S_2_(003) facet model. Reproduced with permission [225]. Copyright 2018, Elsevier B.V

Benefitting from the low cost, excellent conductivity, and considerable electrocatalytic activity, metal sulfides, especially nickel sulfides, are explored as bifunctional electrocatalysts for HER/OER [95, 224]. Dong et al. developed the methods of anodization and vapor sulfurization to synthesis nanoporous thin films of Ni_3_S_2_ with exposed {003} facets supported on nickel foil (Ni_3_S_2_ NTFs) [225]. The XRD results illustrated Ni_3_S_2_ catalysts with different sulfurization time possessed {003} facets (Fig. 20a). The optimized Ni_3_S_2_-300 (sulfurization time of 300 s) displayed remarkable electrocatalytic activity toward HER and OER in alkaline electrolyte with a η of 135 and 175 mV at 10 mA cm^−2^, respectively. The corresponding Tafel slope was 75.5 and 101.2 mV dec^−1^. The Ni_3_S_2_/NF presented TOF values of 0.649 and 0.751 s^−1^ at the η of 300 and 400 mV for HER and OER, respectively (Fig. 20b). The Ni_3_S_2_-300 employed as both anode and cathode electrodes could produce 10 mA cm^−2^ at 1.61 V in 1.0 M NaOH electrolyte (Fig. 20c). The superior performance of the Ni_3_S_2_ is due to accessibility of accessible catalytic active sites and mass transfer channels provided by the controllable hollow nanoporous spheres. Remarkably, the DFT studies reveled that Ni_3_-triangles on {003} facets exhibited a lower energy barrier of 0.93 eV toward water dissociation than S sites, which can be considered as more active for O–H bond cleavage during the Volmer step. However, the S atoms demonstrated smaller ΔG_H_ value compared to that of Ni_3_-triangles, illustrating that S sites were more appropriate for the generation of molecular hydrogen during the Tafel step (Fig. 20d, e). The theoretically result unveiled Ni_3_-triangles of Ni_3_S_2_ {003} facets can boost water dissociation dynamics. For OER, the major cause of high catalytic performance is related to the combined effects of Ni_3_S_2_ and NiOOH.

In another work, Feng et al. constructed Ni_3_S_2_ nanosheet arrays dominated by high-index {${\overline{2}}10$} facets and low-index {001} facets on foam nickel (Ni_3_S_2_/NF) via the direct sulfurization method [92]. The nanosheet array architecture and highly active {${\overline{2}}10$} facets of the Ni_3_S_2_/NF guaranteed the distinguished HER and OER catalytic activity in 1.0 M NaOH with a η of 223 and 260 mV to yield the cathodic and anodic current densities of 10 mA cm^−2^, respectively. The electrolyzer assembled by Ni_3_S_2_/NF as both anode and cathode could produce a water splitting current density of about 13 mA cm^−2^ at approximately 1.76 V. The DFT computations were performed to understand the surface structures and the catalytic activity of {${\overline{2}}10$} and {001} facets. The R ring site containing two Ni5 sites on the terrace of {${\overline{2}}10$} facets were the most active site and possessed the lowest Δ*G*_H_ of 0.496 eV, indicating the high catalytic activity of the R ring site toward HER. The DFT results also disclosed that {${\overline{2}}10$} facets required a smaller η of 0.58 V compared to the value of {001} facets (0.70 V), demonstrating better catalytic activity of {${\overline{2}}10$} facets toward OER.

## Summary and Outlook

**Fig. 21:**
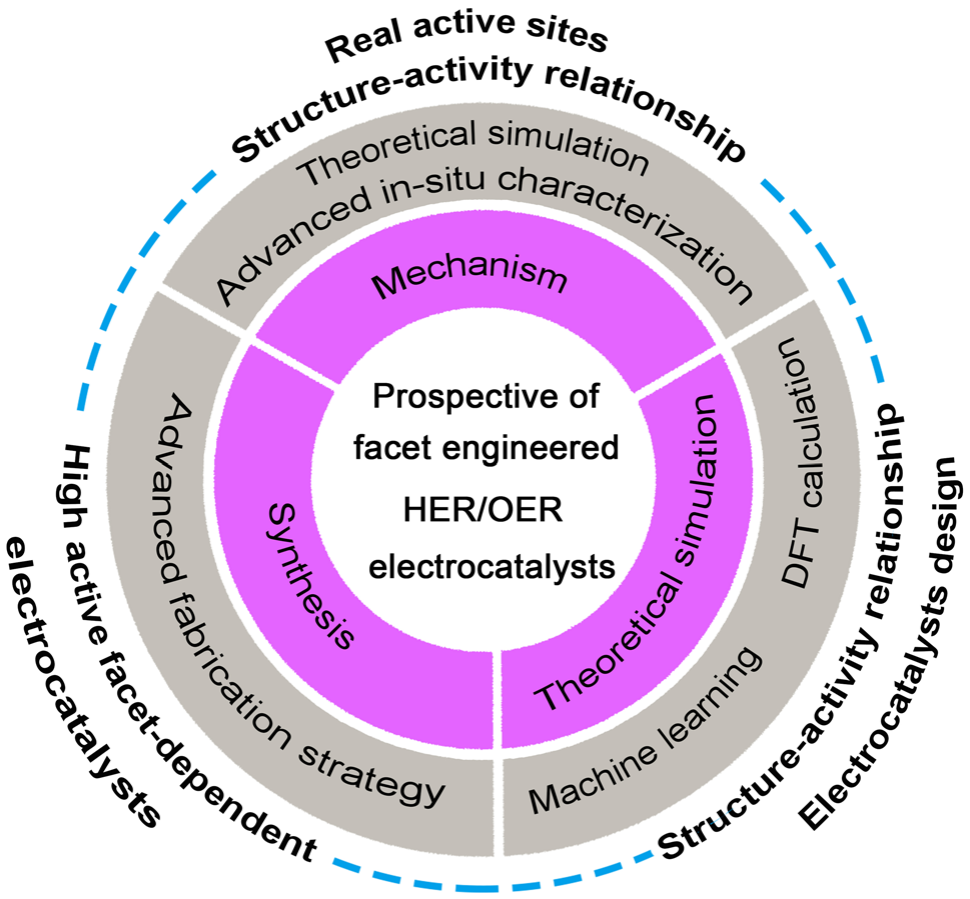
Schematic diagram of the future development of facet-engineered HER/OER electrocatalysts

Benefitting from the advantages of high energy density, natural abundance, and clean combustion, hydrogen is considered as the ideal clean fuel capable of solving global energy crisis and environmental issues and thus realizing carbon neutrality. Electrocatalytic water splitting provides an effective route to produce high-purity hydrogen with zero environmental pollution. The crystal facets featured with facet-dependent physical and chemical properties would exhibit different catalytic activity and selectivity toward HER and OER attributed to their anisotropy. Crystal facet engineering can control the percentage of target crystal planes on the entire crystal surface. Therefore, tailoring crystal planes by crystal facet engineering and further exposing dominant crystal planes with high activity is an effective strategy, which is beneficial for constructing and exploring high-performance OER/HER catalysts. In this review, we firstly outlined the basic concepts, fundamental mechanisms, and evaluation parameters of HER and OER. Then, we summarized the formation mechanisms of the targeted crystal facets and introduced various strategies (selective capping agent, selective etching agent, and coordination modulation) to elaborately control crystal planes. Besides, we also summarized the significant contributions of facet-engineered catalysts toward HER, OER, and OWS. In particular, we highlighted that the DFT calculations played an indispensable role in explaining the superior electrocatalytic activity of dominant crystal planes. Furthermore, some typical examples of facet-engineered electrocatalysts for HER, OER, and OWS are summarized in Table 2. As discussed above, although exciting advancements have been made in facet-engineered electrocatalysts for HER, OER, and OWS, some challenges still exist in the following aspects (Fig. 21):

**Table 2:** Summary of facet-engineered catalysts for the HER, the OER, and the OWS

Catalysts	Substrates	Electrolyte/reactions	Dominant facets	Overpotential *η* (mV) at 10 mA cm^−2^	Tafel slope (mV dec^−1^)	References
Pt NDs	Glassy carbon	0.5 M H_2_SO_4_/HER	{111}	27	22.2	[161]
Raspberry-like SbPt NPs	Glassy carbon	0.5 M H_2_SO_4_/HER	{110}, {100} {101}, {012}	27	50.5	[162]
Pt NSs/CNTs	Glassy carbon	1.0 M KOH/HER	{311}, {200},{111}	36	44	[160]
Ni_1.8_Cu_0.2_–P/NF	Ni foam	1.0 M KOH/HER	{201}	78	70	[226]
CoP UPNSs	Glassy carbon	0.5 M H_2_SO_4_/HER	{200}	56	32	[227]
Ni_5_P_4_ MBs	Ti foil	0.5 M H_2_SO_4_/HER 1.0 M KOH/HER	{001}	35.4 47	48 56	[165]
Ni_2_P	Ti foil	0.5 M H_2_SO_4_/HER	{001}	130@20	30	[167]
Ni_5_P_4_–Co_2_P/NCF	Ni–Co alloy foam	1.0 M KOH/HER	{303}	21	23	[170]
Ni_2_S-octa	Glassy carbon	1.0 M KOH/HER	{111}	138	139	[82]
Meso-FeS_2_	Ni foam	0.1 M KOH	{210}	96	78	[110]
V_2_Se_9_@PEDOT NSs/NF	Ni foam	0.5 M H_2_SO_4_/HER	{100}	72	36.5	[228]
V_8_C_7_@GC NSs/NF	Ni foam	1.0 M KOH/HER 0.5 M H_2_SO_4_/HER 1.0 M PBS/HER	{110}	49 38 77	44.5 34.5 64	[178]
TaC NCs@C	Glassy carbon	0.5 M H_2_SO_4_/HER	{222}	146	143	[94]
GaN	GaN self-made electrode	1.0 M KOH/HER 0.5 M H_2_SO_4_/HER	{100}	171 168	45 36	[73]
Pd@Ir TOH	Glassy carbon	0.1 M HClO_4_/OER	{331}	300	84.9	[191]
Ag_2−*x*_O/FTO-1	FTO	0.1 M K_2_B_4_O_7_/OER	{111}	417	47	[192]
α-Fe_2_O_3_	Carbon fiber paper	1.0 M NaOH/OER	{012}-O	305	51.8	[70]
MnO polypods	Glassy carbon	0.1 M KOH/OER	{100}	580	149	[229]
CoMoO_4_ NR	Glassy carbon	1.0 M KOH/OER	{100}	550@8.93	72	[90]
FeVO_4_ nanobelts	Ni foam	1.0 M KOH/OER	{010}	240	37.4	[195]
CO-Fe_2_O_3_	Glassy carbon	0.1 M KOH/OER	{206}, {119}	439	99	[194]
NiO nanobelts	Ni foam	1.0 M KOH/OER	{110}	382@50	142.5	[193]
CoSe_2_-250	Glassy carbon	1.0 M KOH/OER	{001}	240	75.8	[198]
NiSe_2_	Glassy carbon	1.0 M NaOH/OER	{200}	310	71	[199]
NiFe NSAs-MPs	Ni foam	1.0 M KOH/OER	{012}	250@100	34.5	[207]
Ni_3_Ge_2_O_5_(OH)_4_ nanosheets	Glassy carbon	1.0 M KOH/OER	{100}	320	67.5	[206]
NiFe-MOF NSs	Glassy carbon	1.0 M KOH/OER	{001}	240	73.44	[84]
ZIF-67 (002)	Glassy carbon	1.0 M KOH/OER	{002}	305	67	[80]
Co_3_O_4_ nanooctahedron	Ni foam	1.0 M KOH/OWS	{111}	370	/	[221]
NiCo_2_O_4_ nanosheet	Ni foam	1.0 M KOH/OWS	{111}	360	/	[223]
NiMoN nanowire	Ni foam	1.0 M KOH/OWS	{100}	270	/	[230]
Ni_3_S_2_-300	Ni foam	1.0 M NaOH/OWS	{003}	390	/	[225]
Ni_3_S_2_/NF	Ni foam	1.0 M KOH/OWS	{${\overline{1}}11$}	~ 320	/	[95]
Ni_3_S_2_/NF	Ni foam	1.0 M NaOH/OWS	{${\overline{2}}10$}	590@13	/	[92]
TiO_2_@Ni_3_S_2_	Ni foam	1.0 M KOH/OWS	{${\overline{2}}10$}	350	/	[224]

Firstly, thus far, there are limited strategies to tailor crystal facets and reported strategies possess inherent disadvantages. For the first and second strategies (selective capping agent and selective etching agent) to control planes, the selective effect between the added reagents and the targeted crystal planes is the prerequisite. Worse yet, the strategy of coordination modulation is currently only applicable to MOFs. Additionally, the variety of water splitting catalysts designed by crystal facet engineering is far from abundant to realize industrial hydrogen production. The design and exploration of water splitting catalysts can be beneficial to simplify electrolyzer design, avoid the mismatch of electrolyte, and reduce the cost in device fabrication. Therefore, more attentions should be paid to developing advanced crystal facet control strategies for the construction of promising water splitting catalysts with highly active facets.

Secondly, although high-index facets present satisfactory catalytic performance toward HER/OER, challenges still exist for the preparation of high-index facets. High-index facets usually exhibit a high ratio of low-coordinated atoms, edges, kinks and steps, providing more highly electrocatalytic active sites. However, high-index facets are unstable and often evolve and disappear quickly because of their high surface energy. Accordingly, the design and exploration of new synthetic methods or new capping and etching agents for tailoring the crystal growth process aiming to preserve high-index facets are crucial and highly urgent.

Thirdly, in spite of amazing catalytic performance toward HER/OER for facet-engineered catalysts, the catalytic mechanism and active sites involved in facet-engineered catalysts are still unclear during the HER/OER process. In situ/operando characterization technologies, such as in situ PXRD, in situ XPS, in situ Raman, in situ TEM, and in situ XAS, can track the evolving behavior during HER/OER process, especially the generation and disappearance of hydrogen or oxygen intermediates, and unveil the real catalytic sites, which contributes to determining the HER/OER mechanisms and further uncover the association between the crystal plane and catalytic activity. Unfortunately, a single in situ/operando technique currently cannot offer a complete picture during the dynamic evolution of HER or OER. Accordingly, the collaborative combination of these techniques is of significant for effectively integrating experimental information.

Fourthly, although the arrangement structure and coordination microenvironment of atoms on the crystal plane can greatly affect electrocatalytic activity, the elaborate design of highly active facet-dependent catalysts still faces following challenges: (1) how to adjust the arrangement structure and coordination microenvironment of atoms on the crystal plane at the molecular level to improve inherent catalytic performance; (2) establishment of the structure–activity relationship between the dominant crystal plane and its macroscopic electrocatalytic performance. Intriguingly, the DFT calculation is beneficial to predict the trend in catalytic activity, indicate the real active sites as well as reveal the structure–activity correlations between the dominant crystal plane and electrocatalytic activity. More significantly, owing to the difference between real-world conditions and ideal world conditions for the proposed theoretical structural model, inconsistency between simulation results and experimental results is still unavoidable. Therefore, calling for more attention focus on develop high-efficiency computer software and further design advanced theoretical models of DFT computation, which is beneficial for addressing the above-mentioned challenges and providing valuable principles to the scientific design of high-performance electrocatalysts.

Last but not the least, it is time-consuming and labor-intensive to prepare/screen highly active facet-dependent catalysts from experiments. The machine learning (ML) has emerged as a powerful artificial intelligence (AI) tool capable of data mining and data analysis, and thus providing valuable insights on electrocatalyst design and performance evaluation. The ML featured with powerful data-processing capabilities guarantees the low cost and high efficiency during the process of screening the high-performance electrocatalyst. More importantly, the ML can predict electrocatalytic activity with an accuracy close to DFT. Accordingly, with the help of the ML, constructing the model and further simulating the electrochemical process of active crystal faces are expected to reveal the structure–activity relationship between active facets and electrochemical performances. More effort should be devoted to developing the advanced AI technologies intending to rationally designing facet-dependent electrocatalysts with high activity.
